# Bromodomain and extraterminal proteins foster the core transcriptional regulatory programs and confer vulnerability in liposarcoma

**DOI:** 10.1038/s41467-019-09257-z

**Published:** 2019-03-22

**Authors:** Ye Chen, Liang Xu, Anand Mayakonda, Mo-Li Huang, Deepika Kanojia, Tuan Zea Tan, Pushkar Dakle, Ruby Yu-Tong Lin, Xin-Yu Ke, Jonathan W. Said, Jianxiang Chen, Sigal Gery, Ling-Wen Ding, Yan-Yi Jiang, Angela Pang, Mark Edward Puhaindran, Boon Cher Goh, H. Phillip Koeffler

**Affiliations:** 1 Cancer Science Institute of Singapore, National University of Singapore, Singapore, 117599 Singapore; 2 School of Biology and Basic Medical Sciences, Soochow University, 215123 Suzhou, China; 3 Department of Pathology, UCLA Medical Center, University of California, Los Angeles, CA 90095 USA; 4 Holistic Integrative Pharmacy Institutes, School of Medicine, Hangzhou Normal University, 311121 Hangzhou, China; 5 Department of Medicine, Cedars-Sinai Medical Center, Los Angeles, CA 90048 USA; 6 National University Cancer Institute, National University Hospital, Singapore, 119074 Singapore; 7 Division of Musculoskeletal Oncology, University Orthopaedics, Hand and Reconstructive Microsurgery Cluster, National University Hospital, Singapore, 119074 Singapore; 8 Department of Hand and Reconstructive Microsurgery, National University Hospital, Singapore, 119074 Singapore; 9 Department of Pharmacology, Yong Loo Lin School of Medicine, National University of Singapore, Singapore, 117600 Singapore

## Abstract

Liposarcomas (LPSs) are a group of malignant mesenchymal tumors showing adipocytic differentiation. Here, to gain insight into the enhancer dysregulation and transcriptional addiction in this disease, we chart super-enhancer structures in both LPS tissues and cell lines. We identify a bromodomain and extraterminal (BET) protein-cooperated FUS-DDIT3 function in myxoid LPS and a BET protein-dependent core transcriptional regulatory circuitry consisting of FOSL2, MYC, and RUNX1 in de-differentiated LPS. Additionally, SNAI2 is identified as a crucial downstream target that enforces both proliferative and metastatic potentials to de-differentiated LPS cells. Genetic depletion of BET genes, core transcriptional factors, or SNAI2 mitigates consistently LPS malignancy. We also reveal a compelling susceptibility of LPS cells to BET protein degrader ARV-825. BET protein depletion confers additional advantages to circumvent acquired resistance to Trabectedin, a chemotherapy drug for LPS. Moreover, this study provides a framework for discovering and targeting of core oncogenic transcriptional programs in human cancers.

## Abstract (web summary)

Liposarcoma (LPS) is a rare cancer that can acquire resistance to chemotherapy. Here, the authors map super-enhancers in LPS, finding BET-protein dependent mechanisms that can be targeted by a BET protein degrader, which also can overcome acquired resistance to chemotherapy in LPS.

## Introduction

Transcription factors (TFs) coordinate the expression of target genes typically through cis-regulatory DNA elements. A small set of lineage-specific master TFs and/or de novo chimeric fusion TFs dictate the core transcriptional programs governing cell identity and malignant state^1^. Elucidating the core transcriptional regulatory mechanisms is necessary to understand the fundamentals of molecular carcinogenesis.

Liposarcomas (LPSs) are a group of mesenchymal malignancies showing adipocytic differentiation and are the prevailing types of soft tissue sarcomas in adults^2^. LPSs are heterogeneous diseases with four major subtypes: well-differentiated LPS (WDLPS), de-differentiated LPS (DDLPS), myxoid LPS (MLPS), and pleomorphic LPS (PLPS). The latter three comprise the majority of high-grade cases. DDLPS and PLPS are largely refractory to current treatment modalities, while MLPS shows generally better clinical response and prognosis^3–5^. Although recent approval of Trabectedin (Yondelis) for LPS treatment offers a new option of systematic chemotherapy agent, durable benefits are hampered by clinical toxicity, unresponsiveness, and acquired resistance^6,7^. Unfortunately, local recurrence and distant metastasis occur frequently in advanced LPSs^8^, urging the development of novel therapeutic interventions.

Seminal studies reveal comprehensively somatic abnormalities within LPS genomes^3,9–11^. Amplification of chromosome 12q13-15 and overexpression of CDK4 and MDM2 are prevalent in WDLPS and DDLPS patients, which has guided clinical investigation of MDM2 and CDK4 inhibitors^12,13^. Genomic rearrangements involving FUS-DDIT3 and EWSR1-DDIT3 translocations define MLPS subtype, which shows the highest response rate and survival benefit from Trabectedin treatment^14–17^. Trabectedin binds to the minor groove of the DNA double helix and impairs DNA repair and transcription processes, resulting in growth arrest, differentiation, and cell death^18^. Trabectedin induces maturation of lipoblasts via inactivation of FUS-DDIT3 in MLPS^19,20^. Aberrant DNA methylation and histone modifications have also been implicated in liposarcomagenesis^3,11,21^. Promoter hyper-methylation silences the expression of master pro-adipogenic TFs: CEBPA and KLF4^3^. Increase of H3K9me3 is associated with de-differentiated phenotype and repression of KLF6^21^. To date, tremendous efforts have been made to determine genomic and epigenetic defects that block terminal differentiation of high-grade LPS, whereas the feed-forward transcriptional regulatory mechanism that reinforces and stabilizes the malignant characteristics remains unexplored.

Super-enhancers (SEs) are recognized as active and clustered enhancers that acquire excessive transcriptional machinery and permissive chromatin marks (e.g., H3K27ac)^22^. SE-driven genes are often associated with disease-related oncogenes and lineage-specific master regulators^22,23^. As little is known about enhancer dysregulation in liposarcomagenesis, uncovering the SE architectures will be important to improve the current understanding of epigenetic mechanism underlying LPS malignancy. SE regions are bound asymmetrically by BRD4, one of the bromodomain and extraterminal (BET) family proteins that read histone lysine acetylation and co-activate key oncogenic transcription^23,24^. To date, although BET bromodomain inhibitors (BBIs) have been shown extensively to disrupt the SE activity and display promising anti-cancer effects^25^, the function of BET proteins and their druggability in LPS are still unexplored.

The current study was designed to elaborate the BET protein dependency and its mechanistic connections to the aberrant enhancer states and core transcriptional programs in LPS. We demonstrate that (1) BET proteins are vital to maintain the DDLPS-specific core transcriptional regulatory circuitry consisting of SE-associated TFs FOSL2, MYC, and RUNX1; and (2) BRD4 is a novel co-activator for FUS-DDIT3 function in MLPS. We also report the superior anti-LPS efficacy of BET protein-degrading agents, which provides important insights to targeted depletion of BET proteins as a candidate therapeutic approach for LPS.

## Results

### Charting the super-enhancer landscape in DDLPS and MLPS

**Fig. 1:**
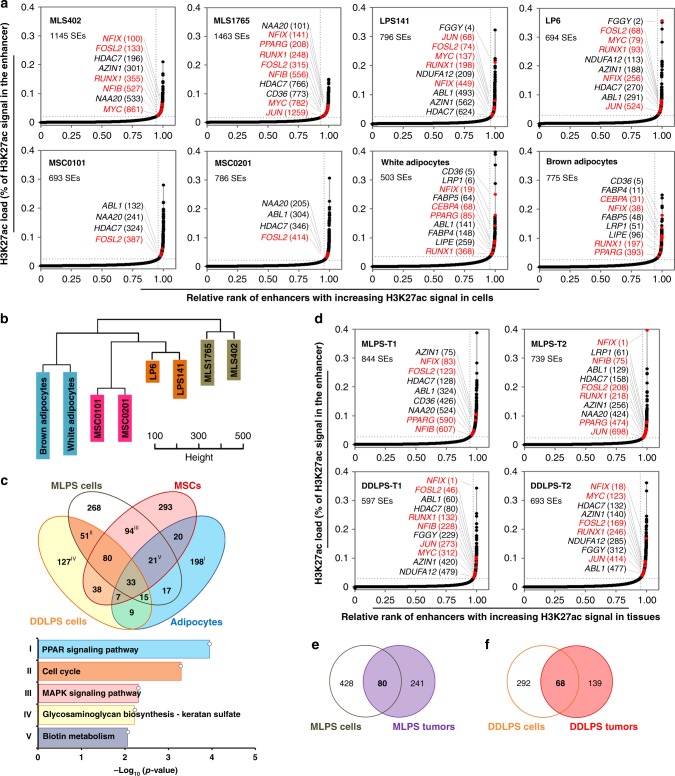
Super-enhancer profiling in de-differentiated LPS (DDLPS) and myxoid LPS (MLPS). **a** Relative rank of stitched H3K27ac ChIP-seq signals in adipocytes, mesenchymal stem cells (MSCs), DDLPS (i.e., LPS141 and LP6) cells, and MLPS (i.e., MLS402 and MLS1765) cells. Representative super-enhancer (SE)-associated genes and their rankings in parentheses were highlighted. Transcription factors were marked in red. **b** Unsupervised clustering of SE regions across the eight cell lines. Colors represent cell types. **c** Venn-diagram showing common and cell-type-specific SE-associated genes across eight cell line samples. SE-associated genes that were present commonly in same cell types were compared. Lower panel indicates top KEGG pathways by which genes from indicated categories were overrepresented (hypergeometric test). **d** Relative rank of stitched H3K27ac ChIP-seq signals in primary DDLPS and MLPS tissues. **e**, **f** Venn-diagram showing common and sample type-specific SE-associated genes across (**e**) MLPS and (**f**) DDLPS. SE-associated genes that were present commonly in either cell lines or fresh tumors of same subtype were compared. Source data are provided as a Source Data file

To evaluate the active epigenetic states associated with LPS malignancy, we performed chromatin immunoprecipitation followed by next-generation sequencing (ChIP-seq) of histone mark H3K27ac in both LPS cell lines and primary tumors. We first compared the H3K27ac-inferred SE architectures in mesenchymal stem cells (MSCs)^26^, mature adipocytes^27^, and cells derived from MLPS and DDLPS (Fig. 1a and Supplementary Fig. 1). SE-association captured the vast majority of highly expressed genes, which can distinguish differentiation stages, disease states, and subtypes of these cells (Fig. 1b and Supplementary Fig. 2a–c). LPS cells engaged preferentially SEs to genes involved in cancer pathways, as well as biological processes of development, cell migration, angiogenesis and transcription from RNA-Pol2 promoter (Supplementary Fig. 2d, e). In contrast, SE-associated genes that were exclusive to adipocytes (e.g., *CEBPA* and *PPARG*) were enriched in PPAR signaling pathway (Fig. 1c and Supplementary Table 1). Of note, we also mapped the SE structures in primary tumors and found that MLPS and DDLPS tissues had 25% and 33% of SE-associated genes overlapping, respectively, with LPS cell lines of the same subtype (Fig. 1d–f and Supplementary Table 2). Altogether, these data are insightful for molecular pathogenesis of LPS, and motivate us to explore the disease-specific gene regulation in MLPS and DDLPS.

### Occupancy of FUS-DDIT3 across super-enhancers in MLPS

**Fig. 2:**
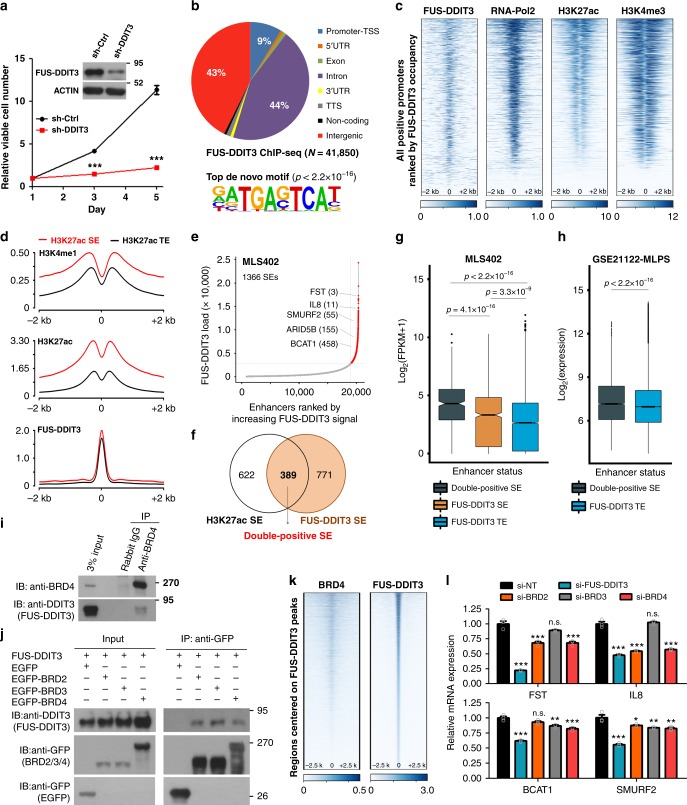
Disproportionate occupancy of FUS-DDIT3 across myxoid LPS (MLPS) genome. **a** Effect of FUS-DDIT3 silencing on cell viability of MLS402 cells. Data are presented as mean ± SEM; *n* = 3. Two-tailed Student’s *t*-test was used. **b** Pie chart showing the genomic occupancy of FUS-DDIT3 peaks in MLS402 cells. Top de novo DNA-binding motif of FUS-DDIT3 was identified by Homer (hypergeometric test). TSS, transcription start site; UTR, untranslated region; TTS, transcription termination site. **c** Heatmaps for the ChIP-seq signals of indicated antibodies ± 2 kb from TSS in MLS402 cells. **d** Differential enrichment of FUS-DDIT3 in SE and typical enhancer (TE) regions. **e** Rank order of stitched FUS-DDIT3 ChIP-seq signals in MLS402 cells. Representative FUS-DDIT3-overloaded genes and their rankings were highlighted. **f** Venn-diagram showing number of genes with their SEs overloaded with H3K27ac and/or FUS-DDIT3 in MLS402 cells. **g**, **h** FUS-DDIT3/H3K27ac double-positive SEs were associated with high basal gene expression in **g** MLS402 and **h** primary MLPS samples (*n* = 20). Wilcoxon signed-rank test was applied. FPKM, fragments per kilobase million. Box plots indicate median value (center line), first and third quartiles (box limits), as well as minimum and maximum values (whiskers) after excluding outliers (dots). **i** Co-immunoprecipitation (IP) between endogenous BRD4 and FUS-DDIT3 in MLS402 cells. **j** GFP-IP showing the interaction between FUS-DDIT3 and BET proteins. FUS-DDIT3 was co-expressed with either EGFP or EGFP-tagged BET proteins in HEK293T cells. **k** Co-localization of FUS-DDIT3 and BRD4 across the genome of MLS402 cells. Heatmaps were used to present the ChIP-seq signals of indicated antibodies ± 2.5 kb from the peak centers of FUS-DDIT3 in MLS402 cells. **l** Quantitative reverse transcription PCR (qRT-PCR) analysis showing the mRNA levels of FST, IL8, BCAT1, and SMURF2 upon small-interfering RNA (siRNA)-mediated knockdown of FUS-DDIT3, BRD2, BRD3, and BRD4, relative to si-NT. RNA was harvested 48 h post transfection in MLS402 cells. Data are presented as mean ± SEM; *n* = 3. Significance was reported within each target gene based on one-way analysis of variance (ANOVA). n.s., not significant; *p < 0.05; **p < 0.01; ***p < 0.001. Source data are provided as a Source Data file

The preferential association of SEs to genes regulating RNA-Pol2 activity prompted us to interrogate potential transcriptional addiction. MLPS manifests core dependency on FUS-DDIT3 (Fig. 2a and Supplementary Fig. 3a–d). Genome-wide occupancy analysis indicated that about 9% of FUS-DDIT3 peaks bound active promoters with broader RNA-Pol2 loading and higher transcription (Fig. 2b, c and Supplementary Fig. 3e, f). Moreover, nearly 60% of FUS-DDIT3 peaks were mapped to putative enhancers, including 97% of H3K27ac-defined SEs and 62% of typical enhancers. Interestingly, the intensity of FUS-DDIT3 peaks was enhanced moderately but significantly inside SEs, coincided with elevated permissive histone marks (Fig. 2d and Supplementary Fig. 3g). FUS-DDIT3 loading in SE was strongly associated with low promoter-proximal pausing of RNA-Pol2 in target genes (Supplementary Fig. 3h). By stitching the peaks of FUS-DDIT3, we discovered FUS-DDIT3-overloaded enhancers and found that genes associated with H3K27ac/FUS-DDIT3 double-positive SEs (e.g., *FST* and *IL8*) showed high basal expression in MLPS (Fig. 2e–h). Next, by interrogating SEs in primary MLPS tissues, we identified a list of genes, including *SMURF2* and *ARID5B* as potential FUS-DDIT3 targets whose expression were regulated through H3K27ac/FUS-DDIT3 double-positive SEs (Supplementary Tables 2 and 3). As BRD4 has displayed asymmetrical loading in SE regions, we hypothesized that FUS-DDIT3 may function together with BRD4 in regulating expression of SE-associated genes in MLPS. In support of this, we identified a physical interaction between BRD4 and FUS-DDIT3, and demonstrated further BET proteins as novel partners of FUS-DDIT3 (Fig. 2i, j and Supplementary Fig. 3i). Moreover, about 40% of BRD4 ChIP-seq peaks co-localized with FUS-DDIT3 (*n* = 3611) across the MLS402 genome (Fig. 2k). Silencing of BET genes resembled partially the regulatory effect of FUS-DDIT3 knockdown on downstream targets (Fig. 2l). Altogether, our data reveal that FUS-DDIT3 and BET proteins cooperate to regulate expression of SE-associated genes in MLPS, and support the notion that fusion TFs hijack BET proteins for malignant transformation^28^.

### Core transcriptional regulatory circuitry in DDLPS

**Fig. 3:**
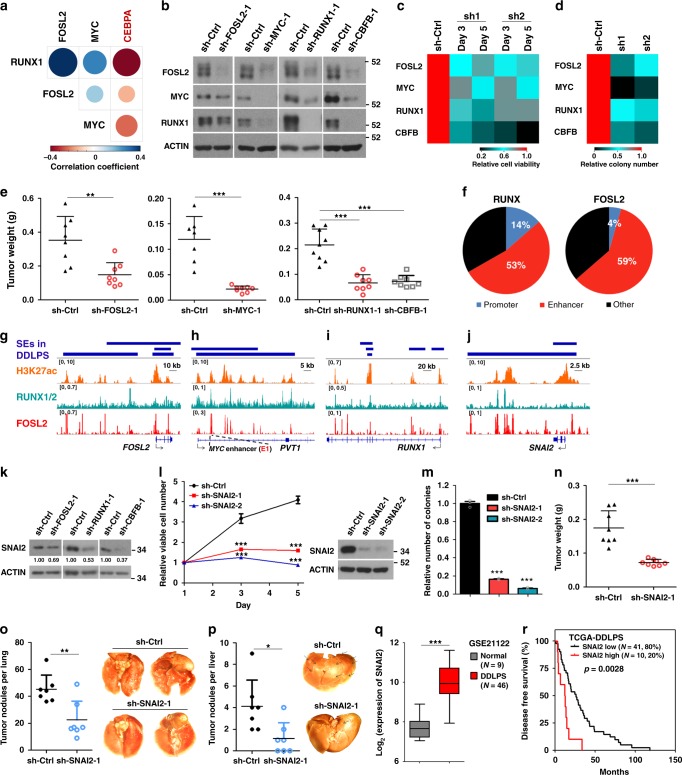
Discovery of core transcriptional regulatory circuitry in de-differentiated LPS (DDLPS) cells. **a** Pearson correlation matrix for the expression values of four TFs based on RNA-seq data from TCGA-DDLPS patient cohort (*n* = 57). RUNX1, FOSL2, and MYC were candidate core TFs in DDLPS. *CEBPA* was included as an example of negatively correlated gene. **b** Effects of core TF and CBFB knockdown on the protein expression of indicated TFs. Whole-cell lysates were extracted from LPS141 cells stably expressing short hairpin RNAs (shRNAs) against respective genes. **c**–**e** Effects of shRNA-mediated silencing of core TFs and CBFB on **c** cell viability, **d** anchorage-independent growth, and **e** tumorigenic ability of LPS141 cells. **f** Pie charts of RUNX and FOSL2 binding to cis-regulatory regions of the DDLPS genome. **g**–**j** Co-occupancy of FOSL2 and RUNX proteins across the SEs of **g**–**i** core TF genes and **j** *SNAI2*. E1 inside the intron 1 of *PVT1* locus has been well-studied as *MYC* enhancer (see also Supplementary Fig. 4g). **k** Effects of FOSL2, RUNX1, and CBFB knockdown on protein level of SNAI2 in LPS141 cells. **l**–**p** Effects of shRNA-mediated silencing of SNAI2 in LPS141 cells on their **l** viability, **m** anchorage-independent growth, **n** tumorigenic ability, and distant metastatic potential to **o** lung and **p** liver. Arrows in **p** indicate tumor nodules. **q** Upregulated expression of SNAI2 in DDLPS samples relative to normal fat tissues. Box plot indicates median value (center line), first and third quartiles (box limits), as well as minimum and maximum values (whiskers). **r** Association of SNAI2 expression with disease-free survival time of patients in TCGA-DDLPS cohort (*n* = 51). Log-rank test was applied. Data of **e**, **l**–**p** are presented as mean ± SEM. Student’s *t*-test (two-tailed) was applied in **e**, **l**–**q**. *n* = 3, in **c**, **d**, **l**, **m**; *n* ≥ 7, in **e**, **n**, **o**, **p**. *p < 0.05; **p < 0.01; ***p < 0.001. Source data are provided as a Source Data file

Inspired from MLPS and other cellular system^1,23,24,28^, SE regions are often loaded densely with lineage/disease-specific master trans-acting factors. Next, we sought to identify TFs associated with aberrant SE architectures in fusion-negative-DDLPS. By scanning known TF motifs in the SE regions of LPS141 and LP6 cells, a list of SE-associated TFs with both auto- and mutual- regulatory potentials was identified and subjected to computational reconstitution of feed-forward core transcriptional regulatory circuitry (CRC)^29,30^. Further, SE ranking and co-expression analysis yielded RUNX1, FOSL2, and MYC as top co-operative core TFs in both DDLPS cells and primary DDLPS tissues (Fig. 3a and Supplementary Fig. 4a). Silencing of core TFs diminished expression of each other, which was also observed by depletion of CBFB, the heterodimeric subunit for RUNX proteins (Fig. 3b). Importantly, silencing of individual core TFs and CBFB attenuated cell viability, clonogenic growth and tumorigenicity of DDLPS cells (Fig. 3c–e and Supplementary Fig. 4b–e). To explore downstream network of core TFs, we mapped the genome-wide occupancy of FOSL2 and RUNX proteins (pan-RUNX). Half of FOSL2 and RUNX peaks were located in enhancers, capturing over 90 and 30% of SEs (Fig. 3f and Supplementary Fig. 4f), respectively. Nearly 70% of SE-associated RUNX peaks overlapped with FOSL2 signals, indicative of a collaborative activity of FOSL2 and RUNX proteins. FOSL2 and RUNX proteins co-occupied at the SE regions of all core TF genes (Fig. 3g–i and Supplementary Fig. 4g)^31^. Additionally, SNAI2 was identified as an important downstream target of FOSL2 and RUNX1 to reinforce the proliferative, tumorigenic, and metastatic capabilities of DDLPS cells (Fig. 3j–p and Supplementary Fig. 4h, i). Indeed, SNAI2 transcripts were significantly elevated in DDLPS samples (Fig. 3q). Higher expression of SNAI2 was associated with shorter disease-free survival of DDLPS patients (Fig. 3r). Altogether, these data identify a feed-forward transcriptional program in maintaining DDLPS malignancy.

### Involvement of BET proteins in liposarcomagenesis

BET proteins including BRD2, BRD3, and BRD4 regulate enhancer activity and transcriptional output^23,24^. Inhibition of BET bromodomains has been shown to impair the expression and/or activity of key oncogenic TFs (e.g., MYC, and PAX3-FOXO1)^28,32,33^. Having shown the core dependency of MLPS cells on FUS-DDIT3/BET proteins and DDLPS cells on newly identified CRC, we sought for a strategy to target these oncogenic transcriptional structures. As little is known about BET proteins in LPS, the potential functional interplays between BET proteins and disease-specific core TFs prompted us to explore their roles in liposarcomagenesis and LPS-addicted transcription.

**Fig. 4:**
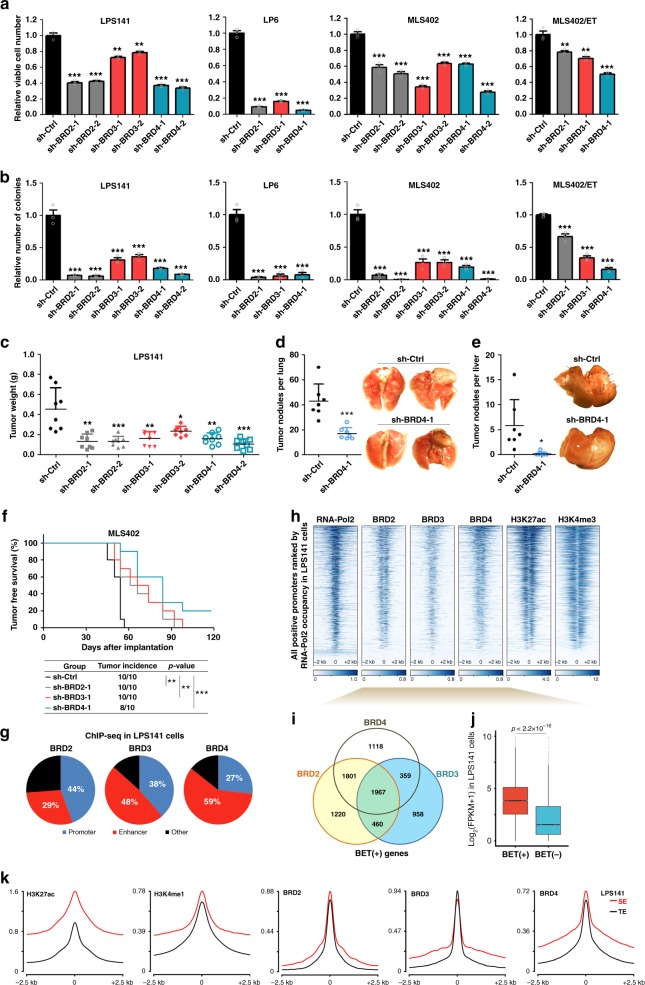
BET proteins maintain the liposarcoma (LPS) malignancy and active transcription. **a**, **b** Effects of short hairpin RNA (shRNA)-mediated silencing of BRD2, BRD3, and BRD4 on **a** cell viability and **b** anchorage-independent growth of DDLPS and MLPS cells. Data are presented as mean ± SEM; *n* = 3. **c**–**e** Effects of shRNA-mediated silencing of BRD2, BRD3, and BRD4 in DDLPS cells on their **c** tumorigenic ability (*n* = 8) and distant metastatic potential (*n* = 7) to **d** lung and **e** liver. Arrows indicate tumor nodules in liver. One-way analysis of variance (ANOVA) was applied for **a**–**c**; Student’s *t*-test (two-tailed) was applied in **d**, **e**. **f** Effect of shRNA-mediated silencing of BRD2, BRD3, and BRD4 on tumorigenic ability of MLPS cells. Tumor-free survival of xenograft-bearing animals and tumor incidence were recorded as the endpoint of experiment. Log-rank test was applied for statistical analysis (*n* = 10). **g** Pie charts of BET proteins binding to cis-regulatory regions of the LPS141 genome. **h** Heatmaps for the ChIP-seq signals of indicated antibodies ± 2 kb from TSS in LPS141 cells. **i** Venn-diagram showing BET (+) genes defined by promoter-proximal occupancy of BET proteins. **j** Differential expression of BET (+) genes and BET (−) genes in LPS141 cells. Box plot indicates median value (center line), first and third quartiles (box limits), as well as minimum and maximum values (whiskers) after excluding outliers (dots). Wilcoxon signed-rank test was applied. **k** Metagene plots showing differential enrichment of BET proteins in SE and TE regions of LPS141 genome. *p < 0.05; **p < 0.01; ***p < 0.001. Source data are provided as a Source Data file

We found that BRD2, BRD3, and BRD4 were expressed prevalently in LPS (Supplementary Fig. 5a), with BRD3 and BRD4 showing significant elevation relative to normal fat tissues. Functional assays demonstrated that BRD2/3/4 were essential for in vitro proliferation of LPS cells, including MLS402/ET, an isogenic line of MLS402, which acquired resistance to Trabectedin^7^ (Fig. 4a, b and Supplementary Fig. 5b–e). Silencing of BET genes also retarded subcutaneous tumor formation in a DDLPS model (Fig. 4c). Knockdown of BRD4 attenuated further distant metastasis (Fig. 4d, e). Similarly, depletion of BET genes prolonged the tumor-free survival of recipient mice in a MLPS model, with BRD4 knockdown reducing both incidence and burden of tumor (Fig. 4f).

To gain insights into the BET dependency, we mapped genome-wide occupancy of BRD2/3/4 proteins across LPS141 genome. BET proteins not only bound active promoters that are positive for RNA-Pol2, H3K4me3 and H3K27ac, but also marked actively transcribed genes, including all core TFs and *SNAI2* (Fig. 4g–j and Supplementary Fig. 5f). Notably, 58% of overall BET-positive promoters were co-occupied by at least two BET proteins. BRD2/3/4 triple-positive genes showed the highest basal expression (Supplementary Fig. 5g, h), suggesting both co-operative and non-redundant functions of BET proteins. Meanwhile, BET proteins distributed asymmetrically to enhancers (Fig. 4k), with all SEs being loaded with at least one BET protein and SE regions of three core TF genes being co-occupied by all three BET proteins (Supplementary Fig. 5i). Hence, the widespread loading of BET proteins in active promoter and SEs implicates their extensive engagement in transcriptional dysregulation in LPS.

### Inhibition of BET proteins exerts potent anti-LPS efficacy

**Fig. 5:**
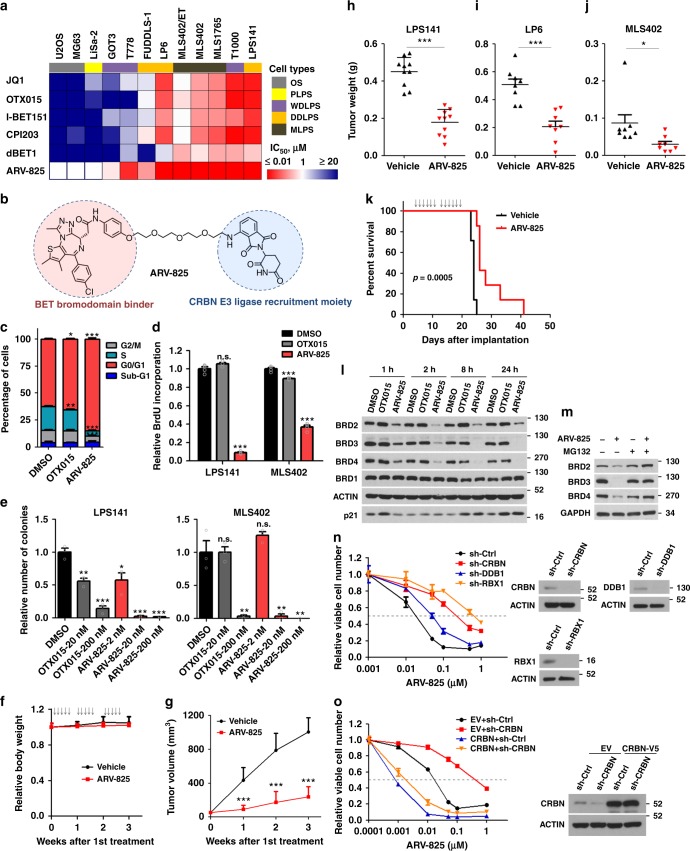
ARV-825 redirects CRL^CRBN^ to disrupt BET protein dependency of liposarcoma (LPS). **a** Heatmaps for the mean IC_50_ values of BET-targeting agents in LPS and osteosarcoma (U2OS, MG63) cell lines. IC_50_ values of ARV-825 in U2OS, MG63, and LiSa-2 cells exceeded the maximum dose (1 µM) and were set as 1 µM for heat map illustration. **b** Chemical structure of ARV-825. **c**, **d** Effects of ARV-825 and OTX015 treatments on **c** cell cycle progression and **d** cellular BrdU incorporation in LPS141 cells (200 nM, 24 h). **e** Dose-dependent inhibition of clonogenicity of LPS cells on soft agar by either ARV-825 or OTX015. **f**–**h** Effect of ARV-825 treatment on **f** animal body weight (*n* = 5), **g** xenograft volume (*n* = 10), and **h** weight of LPS141 xenografts at endpoint (*n* = 10). **i**, **j** Effect of ARV-825 treatment on weight of endpoint xenografts from **i** LP6 and **j** MLS402 (*n* = 8). **k** Effect of ARV-825 treatment on overall survival time of mice-bearing distant metastasis of LPS141 cells (*n* = 7). **l** Temporal effects of ARV-825 and OTX015 (200 nM) on indicated proteins in LPS141 cells. **m** Effect of MG132 on the ability of ARV-825 to deplete BET proteins. LPS141 cells were treated with MG132 (5 µM) and/or ARV-825 (200 nM) for 8 h before harvest. **n** Effects of CRBN, DDB1, and RBX1 silencing on the anti-proliferative efficacy of ARV-825 in LPS141 cells. **o** Ectopic CRBN expression in CRBN-silenced cells reversed insensitivity to ARV-825. Data of **c**–**j**, **n**, and **o** represent mean ± SEM; *n* = 3 in **c**–**e**, **n**, and **o**. One-way analysis of variance (ANOVA) was applied for **c**–**e**; Student’s *t*-test (two-tailed) was applied in **g**–**j**; log-rank test was applied in **k**. Source data are provided as a Source Data file

Next, we determined the sensitiveness of LPS cells to BET protein-targeting agents, including four BBIs (JQ1, OTX015, I-BET151, and CPI203) and two dBETs (dBET1^34^ and ARV-825^35^) (Fig. 5a). Generally, DDLPS and MLPS cells were highly responsive, while osteosarcoma cells were least sensitive. All LPS cells except LiSa-2 showed greater susceptibilities (from fourfold to > 100-fold) to ARV-825 than dBET1 and BBIs. Remarkably, MLS402/ET showed slight increase in IC_50_ of BBIs, yet remained hyper-sensitive to dBETs (Supplementary Fig. 6a). Therefore, consistent with their genetic dependency on BET family genes, LPS cells are highly vulnerable to dBETs.

ARV-825 triggered more prominent inhibition of cell cycle progression, BrdU incorporation, and anchorage-independent growth of LPS cells, relative to equimolar BBI OTX015 (Fig. 5b–e). Importantly, ARV-825 treatment at a well-tolerated dose delayed LPS xenograft development and prolonged the survival of mice bearing distal metastasis (Fig. 5f–k and Supplementary Fig. 6b). These data indicate a strong anti-LPS efficacy and therapeutic potential of ARV-825.

Mechanistically, ARV-825 induced a selective and efficient depletion of BET proteins, in contrast to the competitive interference of BET chromatin loading by OTX015 (Fig. 5l and Supplementary Fig. 6c, d). ARV-825 redirected the Cullin-RING ubiquitin ligase (CRL) CRBN to degrade BET proteins in a proteasome-dependent manner (Fig. 5m). Genetic silencing of CRBN restored growth of LPS cells even in the presence of ARV-825, which was also phenocopied by disruption of the CRL^CRBN^ complex via depletion of either RBX1 or DDB1^36^ (Fig. 5n and Supplementary Fig. 6e–h). Conversely, forced expression of CRBN rendered the cells hyper-responsive to ARV-825 (Supplementary Fig. 6i). Restoration of CRBN level in CRBN-silenced cells reversed the ARV-825 responsiveness (Fig. 5o). Thus, cellular activity of CRL^CRBN^ is a key determinant of ARV-825 efficacy. CRBN serves a promising predictive marker for ARV-825 effectiveness.

### BET degrader disrupts oncogenic transcription

**Fig. 6:**
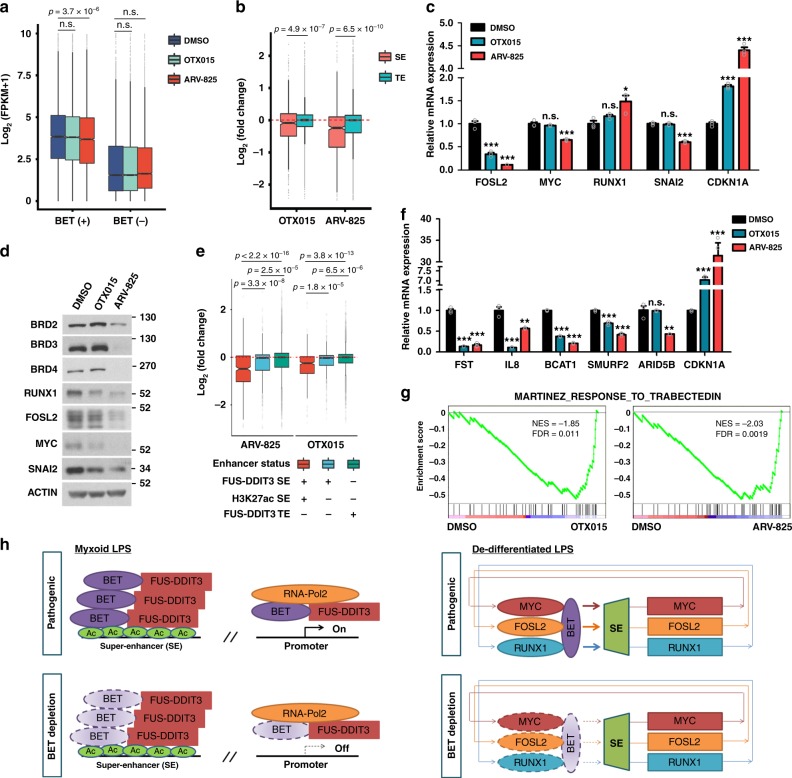
BET protein degrader destroys the core transcriptional programs in liposarcoma (LPS). **a** Differential responses of genes to OTX015 and ARV-825 treatment according to BET protein occupancy around their promoter-proximal regions in LPS141 cells. BET(+) denotes genes with at least one ChIP-seq peak of BET proteins within their promoter-proximal regions; BET(−) denotes genes that are negative for binding of BET proteins. **b** Differential responses of genes to OTX015 and ARV-825 treatment according to enhancer status in LPS141 cells. **c** Quantitative reverse transcription PCR (qRT-PCR) analysis showing the mRNA levels of core TF genes, SNAI2, and CDKN1A upon ARV-825 and OTX015 treatment (200 nM, 24 h) in LPS141 cells. *CDKN1A* was used as positive control for differentially expressed genes. **d** Immunoblot analysis showing the impact of ARV-825 and OTX015 (200 nM, 24 h) on levels of BET proteins, core TFs, and SNAI2 in LPS141 cells. **e** Differential responses of genes to OTX015 and ARV-825 treatment (200 nM, 24 h) according to their enhancer occupancy by FUS-DDIT3 in MLS402 cells. **f** qRT-PCR analysis showing the mRNA levels of FST, IL8, BCAT1, and CDKN1A upon ARV-825 and OTX015 treatment (200 nM, 24 h) in MLS402 cells. *CDKN1A* was used as positive control for differentially expressed genes. Data are presented as mean ± SEM; *n* = 3. **g** GSEA plots of Trabectedin response pathway in MLS402 cells treated with dimethyl sulfoxide (DMSO) versus ARV-825 or OTX015. **h** Models of BET protein-dependent core transcriptional programs in myxoid LPS (MLPS) and de-differentiated LPS (DDLPS). Depletion of BET proteins serves as a common strategy to target these core transcriptional dependencies. Box plots indicate median value (center line), first and third quartiles (box limits), as well as minimum and maximum values (whiskers) after excluding outliers (dots). Wilcoxon signed-rank test was applied for **a**, **b**, **e**; one-way analysis of variance (ANOVA) was applied for **c**, **f**. n.s., not significant; *p < 0.05; **p < 0.01; ***p < 0.001. Source data are provided as a Source Data file

To uncover the transcriptional responses underlying the superior activity of ARV-825 versus OTX015 in LPS cells, a comparative transcriptome analysis was performed. ARV-825 elicited a more pronounced impact than OTX015 on gene expression. ARV-825 down-regulated promoter-BET (+) genes, especially those bound with BRD4, BRD2/4, or BRD2/3/4 (Fig. 6a and Supplementary Fig. 7a). Meanwhile, ARV-825 suppressed preferentially the transcription of SE-associated genes, including *FOSL2*, *MYC*, and *SNAI2*, to a greater extent than OTX015 (Fig. 6b–d). Similar observations were made using dBET6^33,37^, a different BET protein degrader, which has a distinct BET bromodomain binding moiety (Supplementary Fig. 7b–d). Interestingly, while dBET6 can inhibit RUNX1 transcription, ARV-825 reduced the half-life of RUNX1 protein (Supplementary Fig. 7e). These observations also highlight the expressional dependency of core TFs on BET proteins^37^. In MLPS cells, ARV-825 down-regulated selectively the expression of genes associated with FUS-DDIT3/H3K27ac SEs, without affecting FUS-DDIT3 level (Fig. 6e and Supplementary Fig. 7f). Echoing the effects of genetic depletion of BET genes and FUS-DDIT3, we also validated the robust inhibition of FUS-DDIT3 targets by BET-targeting agents (Fig. 6f). Remarkably, both ARV-825 and OTX015 elicited a transcriptional signature of Trabectedin response, which has been attributed to the interruption of FUS-DDIT3 function in MLPS^19,20^ (Fig. 6g), while other chemotherapy drugs, including docetaxel, paclitaxel, and gemcitabine showed no consistent impact on the expression of known Trabectedin-responsive genes (Supplementary Fig. 7g, h). Given the persistent dependency of Trabectedin-resistant MLPS cells on BET proteins, our data provide a strong rationale to circumvent Trabectedin-resistance via chemically induced degradation of BET proteins and suggest a new strategy to inhibit FUS-DDIT3 via scavenging co-operative BET proteins.

## Discussion

In this study, we uncovered a disproportionate genomic distribution of FUS-DDIT3 and its connection to the aberrant transcriptome in MLPS. We have also identified a set of SE-driven transcriptional regulators (RUNX1, FOSL2, and MYC) that collaborate to generate interconnected feed-forward regulatory loops that reinforce the malignancy of human DDLPS. Importantly, both the FUS-DDIT3-driven transcriptional network in MLPS and the core transcriptional regulatory circuitry in fusion-negative DDLPS are maintained by BET proteins, establishing a mechanistic basis for the promising preclinical efficacy of BET-targeting/degrading agents. These data collectively uncover a critical dependency of LPS cells on BET proteins, and provide insights into clinical translation of BET degraders (Fig. 6h).

By charting the first SE landscape in MLPS and DDLPS, our study interrogates the dysregulation of SE and SE-associated genes in liposarcomagenesis. SE-associated genes show a much higher basal expression than those with typical enhancers. Concordant with the phenotypic defect in terminal differentiation, LPS cells retain SEs of genes with known function in early adipogenesis (e.g., *FOSL2*^38^), but ablate SEs associated with adipose-definitive genes (e.g., *CEBPA* and *PPARG*). Albeit the functional outputs of SE and individual enhancers remain controversial^39^, SE analysis helped to identify most genes with high expression and disease-relevance. The de novo SEs in LPS are likely associated with transformation, such as *MYC*, *JUN*, and *CDK6*. Our results encourage a more comprehensive cataloging of enhancers and SEs across LPS subtypes to understand the molecular pathogenesis in this heterogeneous group of diseases.

Although more studies are required to dissect the mechanisms of FUS-DDIT3 in transcriptional activation and repression, the overload of FUS-DDIT3 in SEs implicates its potential function in establishing aberrant epigenetic landscape. Similar to PAX3-FOXO1 in rhabdomyosarcoma and EWSR1-associated fusions in Ewing sarcoma^28,40,41^, our data indicate a substantial co-localization of BRD4 and FUS-DDIT3, and a BET protein-dependent function of FUS-DDIT3 via physical interaction in MLPS. Remarkably, appreciable amounts of EWS-FLI1 and EWS-ERG proteins were found in a large transcriptional complex consisting of BRD4, MED1, and RNA-Pol2 in Ewing sarcoma cells^40^. Either inhibition of BET protein activity or depletion of BET proteins consistently attenuated the fusion-dependent gene expression in the respective sarcoma cells^28,40,42^. However, in contrast to the preferential binding of EWS-FLI1 to GGAA repeats^43,44^, the top binding motif of FUS-DDIT3 resembled that of FOSL2 (AP-1 subunit). Altogether, these observations suggest that BET proteins are commonly hijacked by oncogenic fusion TFs for malignant transformation. As both FUS-DDIT3 and EWSR1-associated fusions involve the N-terminal domains of their respective FET proteins, exploration of whether these fusions show similar dependency on their N-terminus to recruit additional key co-factors such like FOXQ1 and BAF complex would be of interest^43,45^.

CRC stabilizes cell-type/disease-specific transcriptional programs and controls cell identity^46,47^. RUNX1, FOSL2, and MYC formed a CRC in DDLPS cells maintaining their malignant growth. Expression of these core TFs showed a mutually dependent manner. As RUNX family contains three homologous proteins, functional redundancy from other RUNX proteins may also contribute to the transcriptional output of the CRC. In line with this notion, depletion of CBFB phenocopied RUNX1 deficiency, suggesting the engagement of RUNX/CBFB complex in the functionality of CRC. As the functional contribution of MYC to oncogenic transcription has been widely studied, we mainly evaluated the roles of RUNX1 and FOSL2 in DDLPS. ChIP-seq analysis revealed a marked co-occupancy of RUNX proteins and FOSL2 across SEs, supporting their collaborative activity to regulate broad downstream network involved in liposarcomagenesis. We also demonstrated the co-operative function of RUNX1 and FOSL2 in maintaining the expression of SNAI2, a key mediator of epithelial–mesenchymal transition and tumor dissemination^48^. For the first time, our data demonstrate that SNAI2 reinforces proliferative and metastatic capability of LPS cells and that SNAI2 expression is a promising prognostic marker for DDLPS. More studies are still required to understand the activity of SNAI2 and explore its downstream network in promoting DDLPS tumorigenesis. Moreover, BET proteins were essential to foster the expression of core TFs and their downstream targets, echoing recent findings from leukemia^37^. Depletion of BET proteins by two different degraders, ARV-825 and dBET6, inhibited effectively both mRNA and protein levels of MYC, FOSL2, and SNAI2. Both degraders could downregulate RUNX1 protein, although only dBET6 blocked RUNX1 transcription. Persistent RUNX1 transcription under ARV-825 treatment may be associated with unrecognized probe-specific activity/property of ARV-825. Other factors such like gene length may also affect the transcriptional output of downstream targets in response to BET depletion. Despite different mechanisms, both ARV-825 and dBET6 attenuated the expression of RUNX1 protein. Altogether, our data support the concept that BET proteins is a potential Achilles' heel that could be targeted to disrupt the core oncogenic transcriptional programs.

Importantly, our study uncovered the essential roles and desirable therapeutic potentials of BET proteins in LPS. Degradation of BET proteins and inhibition of bromodomains elicited distinct cellular and transcriptional responses, implying differential reliance of LPS cells on BET protein function and bromodomain activity. Consistent with the genetic dependency of LPS cells on BET proteins, depletion of BET proteins by ARV-825 accounted for its superior anti-cancer efficacy. BET protein inhibition/depletion mainly triggered a cytostatic effect in established LPS cell lines, similar to previous studies in established glioblastoma cells and established osteosarcoma cells^33,49,50^. Apart from anti-proliferative activities, BET protein-targeting agents have also been reported to induce apoptosis in a number of cancer cells, including primary osteosarcoma cells^49^, breast cancer cells^51^, and many types of hematopoietic malignancies^35,37,52^. Although we and others have demonstrated that BET protein degraders or inhibitors can effectively suppress the expression of SE-driven MYC, BET protein perturbation had negligible effect on MYC protein in osteosarcoma cells^49^. These heterogeneous responses may be associated with differential intrinsic dependencies on BET proteins and cell-type-specific genomic/transcriptional abnormalities. While our study provides one mechanism to explain the LPS-specific BET protein dependency and its connection to LPS-promoting CRC, main targets of BET proteins are likely different in other cancers lacking FUS-fusions or 12q amplicons. Stubbs et al.^52^ evaluated the BET protein-regulated kinase network in myeloma models, and provided an insightful strategy of combinatory inhibition of BET proteins and their downstream kinases to achieve optimal anti-cancer impact. Hence, BET protein-targeting agents may target distinct downstream molecules in different cancers. Integrative analysis of BET protein-dependent transcriptional network and key other pathways serves as a promising approach to tackle the heterogeneous responses to BET protein-targeting agents in human cancers.

Remarkably, MLPS cells with acquired resistance to Trabectedin remained vulnerable to both genetic and chemical depletion of BET proteins, thus highlighting the potential of targeting BET protein dependency to overcome clinical insensitivity to Trabectedin. In addition, we identified CRBN as a promising predictive marker for ARV-825 efficacy across different sarcoma cells and found alternative factors that may impair ARV-825 efficacy, including co-factor expression and proteasomal activity. Collectively, this study reveals the BET protein-dependent core transcriptional programs that are targetable by BET protein-degrading agents in LPS.

## Methods

### Cell culture

All cell lines tested negative for mycoplasma and are not listed in the ICLAC database. Human embryonic kidney cells 293T (HEK293T, ATCC), U2OS (U-2 OS, ATCC), and MG63 (MG63, ATCC) were cultured in Dulbecco's Modified Eagle Medium (DMEM, Biowest). MLS402 (MLS 402-91), MLS1765 (MLS 1765-92) and GOT-3 cells were generously provided by Dr. Pierre Åman^53–55^. LP6 and LPS141 cells were provided by Dr. Christopher DM Fletcher^56^. T778 and T1000 cells were kind gifts from Dr. Florence Pedeutour. MLS402/ET^7^, FU-DDLS-1^57^, and LiSa-2^58^ cells were gifts from Dr. Eugenio Erba, Dr. Jun Nishio, and Dr. Peter Möller, respectively. All LPS cells were maintained in Roswell Park Memorial Institute medium 1640 (RPMI-1640, Biowest). All aforementioned cell lines were also supplemented with 10% fetal bovine serum (FBS, Biowest) and 1% penicillin-streptomycin (Gibco) in a humidified incubator at 37 °C. All cell lines were authenticated by short tandem repeat analysis with the Geneprint 10 System Kit (Promega). U2OS and MG63 were authenticated with 100% identity to the respective reference cell line in DSMZ database. LPS cell lines reported in this study did not match any known cell lines in the reference databases, including ATCC, DSMZ, Riken, JCRB, and KCLB.

### Plasmids and reagents

All pLKO.1-based short hairpin RNA (shRNA) vectors and lentiCRISPR v2-based sgRNA vectors were listed in Supplementary Table 4. SHC002 was used as non-targeting control (sh-Ctrl). Stable knockdown cell lines were generated by lentiviral infection followed by puromycin (Sigma-Aldrich) selection. pLX304 vector control was a gift from Dr. David Root^59^. pLX304-CRBN-V5 was purchased from DNASU Plasmid Repository. Plasmids expressing GFP-tagged BET proteins^60^, and FUS-DDIT3^61^ were gifts from Drs. Kyle Miller, and David Ron, respectively. FLAG-BRD3 and HA-FUS-DDIT3 constructs were generated in this study via ligation-dependent molecular cloning into pcDNA3.1 (+) vector. SMART-pool small-interfering RNAs (siRNAs) against DDIT3, BRD2, BRD3, BRD4, CRBN, MYC, FOSL2, RUNX1, and CBFB were purchased from GE Dharmacon. ON-TARGETplus Non-targeting Pool (D-001810-10-05) was used as negative control. siRNAs were transfected using RNAiMAX (Life Technologies). The sources of other key chemicals used in this study are listed in Supplementary Table 5.

### Cell viability assay

MTT (3-(4,5-Dimethylthiazol-2-yl)-2,5-diphenyltetrazolium bromide) assay was performed to measure cell viability under indicated treatment^62^. Briefly, cells were seeded into 96-well plates at 2000–3000 cells/well and cultured for specified period. At the end of experiment, MTT substrate (Sigma-Aldrich) was added into each well and incubated for 3 h. After careful removal of medium and addition of 100 µL of MTT STOP solution, plates were shaken on a microplate shaker for 3 h and read on a Tecan microplate reader with the absorbance at 570 nm. IC_50_ values were measured by MTT assay after 96-hour treatment (*n* = 3).

### BrdU incorporation assay

BrdU Cell Proliferation Assay Kit (BioVision Inc.) was used according to the manufacturer's instruction. LPS cells were seeded into 96-well plates at 5000 cells/well and treated with indicated compounds for 24 h. BrdU solution was then added into each assay well and incubated at 37 °C for 3 h before fixation. BrdU incorporated by proliferating cells was detected by an enzyme-linked immunosorbent assay.

### Cell cycle analysis

Cells receiving indicated treatment were trypsinized, washed, and fixed in 70% ethanol at 4 °C. Cells were washed with cold phosphate-buffered saline (PBS), re-suspended in propidium iodide solution containing RNase A, and incubated at 37 °C for 30 min. After measurement by LSR II Flow Cytometer System (BD Biosciences), cell cycle distribution was analyzed by FlowJo software.

### Soft agar colony formation assay

Soft agar colony formation assay was performed to evaluate the anchorage-independent growth of LPS cells with indicated treatment. Basal layer was prepared by adding 500 µL of 0.4% agarose (in RPMI supplemented with 10% FBS) into 12-well plates. After basal layer solidified, 1000–5000 LPS cells were mixed with 500 µL of top layer solution (0.4% low melting agarose in RPMI supplemented with 10% FBS) and dispersed over the basal layer. Plates were placed at 4 °C for 25 min before addition of feeder medium (1 mL) into each well, and then kept at 37 °C in a 5% CO_2_ incubator for 2 weeks. Colonies were stained using 0.01% crystal violet in 4% paraformaldehyde/PBS.

### Animal models

All animal experiments were in compliance with ethical regulations of the NUS Institutional Animal Care and Use Committee. Subcutaneous xenograft model was employed to evaluate the tumorigenicity of LPS cells upon either genetic manipulation or chemical treatment. Indicated amount of LPS cells were mixed with 100 µL of Matrigel (Corning)/PBS solution (volume ratio, 1:1) and injected subcutaneously on the upper flanks of NOD/SCID gamma mice (6- to 8-week-old). For in vivo ARV-825 treatment, mice bearing palpable DDLPS xenografts were randomized into two groups. Mice in experimental arm received ARV-825 at a dose of 5 mg/kg by intraperitoneal (i.p.) injection once a day, 5 days per week; animals in the control arm received the same volume of vehicle (5% Solutol/Kolliphor® HS 15 in PBS). Tumor size was measured by caliper and tumor volume (mm^3^) was estimated according to formula 1/2(Length × Width^2^). Xenografts were dissected and weighed upon harvest.

Experimental metastasis assays via intravenous injection of LPS cells were performed to study genes that regulate metastatic progression. LPS141 cells with indicated genetic manipulation were injected (0.1 million per injection) via tail vein into the NOD/SCID gamma mice (6- to 8-week-old). Three weeks after implantation, recipient mice were sacrificed for examination of metastatic nodules in lung and liver. For the study of ARV-825 treatment in a metastatic model, mice were injected intravenously into the tail vein with 0.5 million of LPS141 cells, and subsequently randomized into two groups. Treatments with either vehicle or ARV-825 (5 mg/kg, once a day, six times per week) were initiated 4-days after cell injection. Animal survival was recorded using 10% loss of peak body weight as a humane endpoint criterion.

No specific randomization method was used to allocate mice into different treatment groups. The investigators were not blinded to allocation during experiments or outcome assessment. Minimum sample sizes for individual experiments were determined not on the basis of a statistical method, but according to experience (*n* ≥ 7). No animal was excluded from these experiments.

### Cell lysate preparation and immunoblot assay

Whole-cell extracts were prepared by incubating cells in lysis buffer (50 mM Tris pH 8.0, 420 mM NaCl, 5% glycerol, 0.1% NP-40, 0.1 mM EDTA) with fresh addition of 1 mM DTT, 1 mM phenylmethylsulfonyl fluoride (PMSF), 1x protease inhibitor cocktail (Roche), 1x phosphatase inhibitor cocktail (Roche), 1 mM MgCl_2_ and Benzonase (1:500, Novagen) for 20 min on ice. To extract chromatin-bound proteins, cell pellet was lysed by CSK buffer (10 mM PIPES, pH 6.8, 300 mM sucrose, 100 mM NaCl, 1 mM MgCl_2_, 1 mM EDTA, 1 mM EGTA, 0.5% Triton X-100) supplemented with protease and phosphatase inhibitors. After centrifugation, non-chromatin soluble fraction was enriched in the supernatant. Insoluble pellet was washed twice with CSK buffer, re-suspended in lysis buffer, sonicated, and digested with Benzonase for 20 min on ice. Clear supernatant after centrifugation (13,000 rpm, 10 min) represented the chromatin fraction. Protein concentration was quantified by Bradford assay. Immunoblot analysis was conducted following standard protocol with indicated antibodies listed in Supplementary Table 6. Uncropped scans of immunoblots are included in the Source Data file.

### Immunoprecipitation (IP)

To prepare cell lysate for either IP or Co-IP, cell nuclei were isolated and lysed in IP buffer (25 mM Tris pH 8.0, 0.15 M NaCl, 2.5% glycerol, 0.05% NP-40, 0.05 mM EDTA) with freshly added 1 mM PMSF, 1x protease inhibitor cocktail, 1 mM MgCl_2_ and Benzonase (1:500, Novagen). M2 Sepharose beads (Sigma-Aldrich), GFP-Trap® beads (ChromoTek), and Pierce® Anti-HA Agarose (Thermo Fisher Scientific) were used for IP of proteins with FLAG-Tag, GFP-Tag, or HA-Tag, respectively. For endogenous IP, one milligram of nuclear extract was incubated overnight with magnetic beads (Invitrogen Dynabeads) conjugated with respective antibodies. Bound proteins were analyzed by sodium dodecyl sulfate polyacrylamide gel electrophoresis (SDS/PAGE) and immunoblot. Primary antibodies for IP assays were listed in Supplementary Table 6.

### Chromatin immunoprecipitation (ChIP) and ChIP-seq analysis

Pathologically reviewed human LPS samples were collected from National University Hospital Tissue Repository. The study of human LPS samples was approved by the NUS Institutional Review Board (reference code N-17-057). ChIP was performed by following a standard protocol. Briefly, either homogenized tissues or cells were fixed with 1% formaldehyde for 10 min at room temperature, followed with three washes using cold PBS. Nuclei were extracted and re-suspended in SDS lysis buffer for 10 min on ice before sonication by Bioruptor (Diagenode). Optimal condition of sonication yielded genomic fragments around 200 to 500 bp. After sonication, cell debris was removed by centrifugation (13,000 rpm, 10 min). Supernatant was then pre-cleared and incubated with magnetic beads (Invitrogen Dynabeads) conjugated with specific antibodies (Supplementary Table 6). After overnight incubation, magnetic beads were washed stepwise with cold low salt wash buffer, high salt wash buffer, LiCl wash buffer, and TE buffer. Bound DNA was eluted, reverse-crosslinked, and purified by QIAquick PCR purification kit (Qiagen). ChIP-seq library was constructed using ThruPLEX® DNA-seq Kit (Rubicon Genomics) following standard protocols, and subjected to Illumina deep sequencing (single-end reads of 50 bases).

For ChIP-seq data analysis, raw reads were aligned to hg19 reference genome using bowtie aligner followed by removal of PCR duplicates with Picard markDuplicates utility^63^. Resulting bam files were used for peak calling with MACS2 by extending reads to 200 bp. ChIP signals (Bedgraph) were simultaneously generated and input signal was subtracted with MACS2 bdgmp function. Bedgraphs were later converted to bigwig files with UCSC bedGraphTobigWig utility. Alignment and peak calling pipelines with hard-coded parameters are available at https://github.com/PoisonAlien/chiptk. Detected peaks were annotated with HOMER annotatePeaks. Denovo motif identification and comparison were also performed with HOMER using findMotifsGenome program. Heatmaps were drawn using deeptools by centering either on identified peaks or on transcription start site (TSS) of known refseq genes^64^. RNA-Pol2 signals had been oriented based on transcription direction in heatmaps or metagene plots where − and + indicated upstream and downstream of TSS, respectively, of genomic regions after alignment with transcription direction. RNA-Pol2 pausing index was estimated based on RNA-Pol2 ChIP-seq data and calculated as a ratio of TSS region signal to gene body signal. For profile plots, signals around +/− 2500 bp of peak center were extracted for every 25 bp bin using bwtool matrix function^65^. Average signal was estimated and plotted in R. ChIP-seq data of adipocytes (GSE59703; sequence length = 50) and mesenchymal stem cells (GSE16256; sequence length = 36) have been published previously.

The ROSE (Rank Ordering of Super-Enhancers) algorithm (https://bitbucket.org/young_computation/rose) was used to call super-enhancers (SEs). Regions within +/− 1250 bp from TSS were excluded for this analysis. Input-subtracted ChIP-seq signals were stitched and ranked based on intensity. A geometrical inflection point was used as cutoff to separate SEs from typical enhancers. SE-association was annotated with Ensemble genes. In Fig. 1a, d, enhancer rank and stitched H3K27ac signals were normalized within each sample. Specifically, *x*-axis was normalized by calculating the Relative enhancer rank; *y*-axis was normalized by calculating the Percentage of H3K27ac signal in enhancer (as indicated by the following equation). Relative enhancer rank = (Enhancer rank of individual stitched enhancer)/(Number of SE and TE identified in each sample). Percentage of H3K27ac signal in the enhancer = 100 × (Sum of H3K27ac signal in each stitched enhancer)/(Sum of H3K27ac signal in all SE and TE). For PCA and hierarchical clustering analysis, ChIP signals for all SEs identified across samples were estimated (area under the peak) followed by log_2_ transformation. PCA was performed with prcomp function. Same data were used for Hierarchical clustering with hclust function. Core transcriptional regulatory circuitries were computationally inferred by CRCmapper^29^ based on scanning of TF motifs within SEs.

The peak distribution of FUS-DDIT3, pan-RUNX, FOSL2, and BET proteins across cis-regulatory regions of the genome was annotated as below: peaks that localized within −1000 to +200 bp from the TSS were defined as Promoter-bound; those present inside either SEs or typical enhancers were defined as Enhancer-bound; remaining peaks were considered as others.

### RNA preparation, qRT-PCR, RNA-seq, and expressional analysis

Total RNA was extracted by RNeasy Kit (Qiagen) and treated with DNase. cDNA library for quantitative reverse transcription PCR (qRT-PCR) was prepared by RevertAid RT Reverse Transcription Kit (Thermo Fisher Scientific). qPCR was conducted with Kapa SYBR fast qPCR Master Mix (KAPA Biosystems) on a 7500 Real-time PCR System (Applied Biosystems). qRT-PCR primers are listed in Supplementary Table 4.

RNA-seq library was constructed by TruSeq Library Prep Kit (Illumina) and sequenced at BGI Tech Solutions Co., Ltd. (Hiseq, paired-end reads of 100 bases). Paired-end reads were pseudo-aligned and quantified to Ensemble (hg19; version 88) transcripts using kallisto program^66^. Kallisto results were imported into DESeq2 using tximport Bioconductor package^67^. Differential gene expression analysis was performed using DESeq2 with lfcThreshold argument set to 0.1^68^. Expression values were calculated in terms of FPKM for every gene with DESeq2::fpkm function. Gene set enrichment analysis (GSEA) was performed on all active genes (mean FPKM > 0.5)^69^.

Transcriptome data for DDLPS samples of Sarcoma cohort (TCGA-SARC, *n* = 57) were downloaded using TCGABiolinks Bioconductor package via GDC (data freeze from Dec-2017). Gene Expression Matrix files for microarray dataset GSE21122^9^ were downloaded using GEOquery Bioconductor package. Gene expression for probes mapping to same genes were averaged to get gene level expression for downstream analysis.

### Statistical analysis

Unless otherwise stated, two-tailed Student’s *t*-test was used to analyze the statistical difference between two groups, while one-way analysis of variance (ANOVA) was applied for multi-group comparison. Log-rank test was used for survival analysis. n.s., not significant; *p < 0.05; **p < 0.01; ***p < 0.001. Sample sizes were not predetermined statistically. Center values, error bars, and number of replicates are described in the corresponding figures and/or figure legends. Replicates represent: (1) separate tumors in xenograft assays; (2) individual animals in experimental metastasis assays, and (3) independent biological repeats for in vitro assays.

### Reporting Summary

Further information on experimental design is available in the Nature Research Reporting Summary linked to this article.

## Supplementary information

Supplementary Information

Reporting Summary

Source Data

## Data Availability

RNA-seq and ChIP-seq data generated in this study have been deposited in NCBI GEO and are available under accession GSE111254. RNA-seq data for DDLPS samples of TCGA Sarcoma cohort are from GDC [https://portal.gdc.cancer.gov/]. cDNA microarray data (GSE21122) of LPS and control normal fat specimens, and ChIP-seq data of adipocytes (GSE59703) and mesenchymal stem cells (GSE16256) are available from NCBI GEO. The source data supporting the findings of this study are available within the article and its Supplementary information files including Source Data file. A reporting summary for this Article is available as a Supplementary Information file.
