# Supplementary material for: Bromodomain and extraterminal proteins foster the core transcriptional regulatory programs and confer vulnerability in liposarcoma
Source: Nat Commun. 2019 Mar 22;10:1353. doi: 10.1038/s41467-019-09257-z (PMC6430783; doi:10.1038/s41467-019-09257-z)
Supplement: Supplementary file 1 — Supplementary Information [file 41467_2019_9257_MOESM1_ESM.pdf]

Supplementary Information

Bromodomain and extraterminal proteins foster the core transcriptional regulatory programs and confer vulnerability in liposarcoma

Chen et al.

Supplementary Table 1: List of adipocyte-specific and LPS cell line-specific SE-associated genes

Groups of SEs[a]	SE-associated genes
**SEs that are exclusive to adipocytes**	*ZNF664, ZFAT-AS1, ZEB2, ZEB1, VKORC1L1, UTRN, USP3, UCP3, TSPAN3, TSPAN15, TSKU, TSEN2, TRAF3IP2, TNS1, TNKS1BP1, TMEM200B, TMEM17, TMEM105, TM2D1, THRSP, TGFBR2, TES, TEF, TECTB, TCP11, TBL1XR1, STRAP, STOM, STK39, STEAP2, STBD1, SP3, SOX4, SOS1, SORT1, SORBS1, SNORA59A, SMURF1, SLC9A1, SLC7A6, SLC5A12, SLC38A9, SLC29A1, SH3PXD2B, SH3GLB1, SH3BP5, SETD1B, SEMA4F, RSPO3, RRAS2, RNLS, RFX2, RFC3, RERE, RAD23B, PTTG2, PTPRF, PSMA1, PROX2, PPTC7, PPP2R5A, PPP2R1B, PPARG, PLIN2, PLA2G4A, PLA2G16, PICALM, PGCP, PDXK, PDE11A, PCSK1, PAX5, PALM2, PABPC1P2, NRBF2, NOD1, NFIA, NEURL, NCRNA00161, NBPF10, MRPS27, MRPL19, MMD, MIRLET7I, MIR499, MIR4305, MIR3945, MIR3165, MIR3119-1, MIR1293, MIR1180, MGST1, MEIS1, MCHR2, LY86-AS1, LSM6, LRP12, LRP1, LOC646982, LOC283392, LOC100526820, LOC100498859, LOC100188949, LOC100129726, LIPE, LGALS12, LAMB3, LAMA4, KLF15, KLB, KBTBD8, KANK1, ICA1, HNRPLL, HK2, HK1, HEXIM1, GRIN2B, GPR109A, GPD1, GPAM, GNAT3, GHR, GALNT12, G0S2, FGF2, FERMT2, FAM193B, FABP5, FABP4, ERV3, ERAP2, EPHX1, EPCAM, ENPP2, ELOVL5, ELMOD3, EHBP1, EED, ECHDC1, EBF1, DSE, DPYD, DMRTA1, DLAT, DIXDC1, DIRC1, DHRS7B, DGKI, DENND2D, DDR2, CYB5A, CXCR7, CSNK1A1, CORO6, CORO2B, COL6A2, COL3A1, COL1A2, COL15A1, CMAH, CLLU1OS, CEBPB, CEBPA, CDH11, CD36, CD200, CCDC36, CAV2, C7orf71, C7orf68, C5orf62, C5orf13, C3orf21, C2orf28, C20orf3, C15orf54, C14orf180, C11orf53, BTBD9, BNIP3L, ATP8B4, ATP10A, ASPN, ARHGAP26, ARHGAP20, AOC3, ANO6, ANKDD1A, ALG9, AKR1C1, AKNA, ADIPOQ, ADCY5, ACSL1, ACACB, ABTB1, ABCA1*
**SEs that are present commonly in DDLPS cells but no other sample types**	*JUN, ISX, IER5L, IBTK, HS3ST3A1, HOXA5, HMP19, HERC4, GREM1, GPC6, GDNF, GAP43, FSTL3, FOXO3, FOXD1, FOSL1, FLJ43681, FGGY, FBXO32, FAM173B, FABP6, ELK3, ELFN1, EDIL3, E2F7, DYRK2, DUSP7, DUSP10, DLX2, DDIT4, CTSB, COL5A1, CLIC4, CHST2, CDKN2B, CDK17, CDA, CCND1, CCIN, CALML3, CACNA1C, BMPR2, BCL6, AZIN1, ASB2, ASAM, ARID1B, ARF6, ANPEP, ADAMTSL1, ABR, ABLIM3, ABHD5*
**SEs that are present commonly in MLPS cells but no other sample types**	*ZYX, ZSWIM6, ZNF827, ZNF655, ZNF469, ZFHX4, ZC3H7B, ZBTB2, XPO6, WDR1, USP44, USP36, TXNRD2, TXN2, TWIST2, TSPAN4, TSPAN2, TRIOBP, TRIM25, TOP1, TNFRSF6B, TMSB10, TMEM212, THSD4, TBC1D23, SYNGAP1, SYNCRIP, SUSD1, SRGN, SRGAP1, SPAG7, SMS, SMAD6, SLC7A5P2, SLC7A5P1, SLC44A3, SLC3A2, SLC16A3, SLAIN2, SIM2, SHC1, SH3TC2, SH3RF2, SETD5, SERPINB2, SDPR, SCOC, SAV1, SARDH, SAMD12, RUNDC2C, RPL26, ROBO4, RNF213, RND3, RIN3, RIC8A, RCC1, RBMS3, RARA, QSOX1, PTRF, PTPRK, PTPRH, PTK2, PTBP2, PSAP, PRR11, PROZ, PRKAG2, PREP, PPP1R15A, POU2F2, PLOD2, PLD1, PIP5K1A, PIM3, PIGV, PHACTR1, PFDN1, PCGF2, PAPD5, PAM, ODZ2, OCLN, NUP153, NRXN3, NMT2, NMNAT2, NGF, NFIB, NEDD4, NDUFV2, NCRNA00275, MYO18A, MUSK, MTSS1L, MRPS24, MRPL36, MRPL33, MIR583, MIR4268, MIR3920, MIR3668, MIR196A1, MIR137, MIR1265, MIR1205, MICALL1, MICALCL, METT5D1, MBNL2, MAX, LYST, LRRC41, LPXN, LPAR1, LOC642852, LOC642587, LOC554202, LOC285593, LOC284751, LOC100310782, LOC100302640, LOC100131496, LOC100128081, LIMA1, LGALS3, LARP6, L3MBTL4, KRT81, KLF2, KIAA1755, KIAA1632, KIAA0427, KCNU1, KCNS1, ITPK1-AS1, ITGBL1, INPP4B, IL8, IL7R, IL6ST, IL6, IL1F7, IGF2BP2, IGF2BP1, HYI, HSPA5, HSF2BP, HS1BP3, HNRNPH3, HNRNPH1, HNRNPF, HNRNPA2B1, HMOX2, HMGA1, HLX, HIST2H3A, HIST2H2BC, HEG1, HCFC1, GYG1, GTF2IRD1, GSK3B, GSG1, GPRC5A, GPR78, GPR176, GPR137, GPC1, GNB1L, GFAP, GALNTL2, FST, FLRT2, FLNC, FJX1, FHOD1, FBLN2, FAM46A, FAM38B, FAM38A, FAM20C, FAM200B, FAM155A, F3, ETS2, ETAA1, EPS8L2, ENG, ENAH, EMP1, EML1, EGFLAM, EBNA1BP2, DYNLRB2, DUSP5, DNAJC7, DNAJB6, DNAJB1, DLGAP4, DLG1, DKK1, DAAM1, CXCL2, CTLA4, CSNK1E, CREBBP, CREB1, CPA3, COQ2, COL16A1, CLIP4, CLIC1, CLEC3B, CHST3, CEBPD, CDH2, CDCA4, CDC42EP4, CD44, CCL2, CCDC85C, CASS4, CAPRIN1, CAMK2D, C9orf27, C9orf150, C7orf69, C7orf51, C6orf223, C2orf18, C22orf26, C20orf72, C20orf117, C1orf144,*

	C14orf70, C11orf71, C11orf68, C10orf54, BTBD3, BNC1, BBS12, BASP1, ATXN2L, ATXN10, ATN1, ATG7, ASXL1, ASB7, ASB1, ASAP1, ARSJ, ARHGAP17, APPL2, AP2M1, ANXA3, ANXA1, ANKRD55, ALDH3B1, AKT3, AKAP6, AHRR, AFAP1, ADRB2, ADORA2B, ABI3BP
**SEs that are present commonly in four LPS cell lines but no other sample types**	*YWHAZ, VEGFC, UACA, TUBB6, TPM4, THBS2, TBX18, SPEN, SMAD3, RPS10P7, RPL22L1, RAI1, PSCA, PFN1, PDP1, PDCD1LG2, PCBP3, NFKBIA, NEDD9, MYEOV, MYC, MIR610, MIR1244-1, MIR1208, MIR100, MGC45800, MEIS2, MATN2, MARCKS, LOC654342, LOC648987, LOC645166, LOC100129316, LHFPL2, KCNMA1, IGF2BP3, ID3, GADD45B, FLNA, EEF1A1, CFLAR, CDK6, BCL3, B4GALT1, ASAP2, ASAP1-IT, ARID1A, APOLD1, ANP32A, ALCAM, ADAMTS6*

[a] This analysis only compared the SEs which occurred commonly in cell lines of same sample type (e.g. DDLPS, MLPS, MSC, and adipocytes).

Supplementary Table 2: List of SE-associated genes which are common in primary LPS tissues and cell lines of respective subtype.

Groups of SEs[a]	SE-associated genes
**SEs that are present commonly in MLPS cells and MLPS tissues**	*ACTN1, ACTN4, AFAP1, AHDC1, AHNAK, ANXA2, ARHGAP23, ARID1A, ARID5B, ATXN1, BCAR1, BTBD19, C10orf54, C6orf223, CEBPD, CFLAR, CHST3, COL1A1, CUEDC1, CXXC5, DUSP1, EFCAB1, EMP1, ENG, EPHA2, ETS1, FAM129B, FBLN2, FOSL2, HDAC7, HEG1, HS1BP3, HSPG2, IRF2BP2, KDM6B, KLF2, KLF7, LAPTM4A, LGALS3, LHFPL2, LMNA, LOXL1, LTBP2, MALAT1, MARCKS, MYH9, MYOF, NAA20, NEAT1, NFIB, NFIX, NR1D1, NR2F2, PDGFRB, PLEC, PTRF, PXDN, PXN, RARA, RASA3, RIN3, RTN4, SAMD4A, SEPT9, SMAD3, SMAD6, SMURF2, SOCS3, SPARC, SSH1, STK40, TCF7L2, TGIF1, THBS2, TNFRSF1A, TNRC18, TRIM8, ZMIZ1, ZMYND8, ZNF217*
**SEs that are present commonly in DDLPS cells and DDLPS tissues**	*ABL1, ACTN4, AHNAK, ANXA2, APOLD1, ARHGEF2, AZIN1, B4GALT1, BCL6, BHLHE40, CFLAR, CHSY1, COL1A1, CTDSP2, CXXC5, DUSP10, FGGY, FOSL2, FOXO3, GADD45B, HDAC7, IER2, IER5L, IRF2BP2, JUN, KLF6, KLF7, KLF9, LHFPL2, LMNA, MALAT1, MARCKS, MEF2D, METRNL, MYC, MYH9, MYOF, NDUFA12, NEAT1, NEDD9, NFIX, NNMT, NOTCH2, NOTCH2NL, NR2F2, NRP1, PLB1, PLEC, PLEKHA2, PLEKHF1, RGS3, RUNX1, SEPT9, SMAD3, SMAD7, SOCS3, SPARC, SSH1, TAGLN2, TGIF1, TNFRSF1A, TNRC18, TRIM8, TTYH3, UBC, ZFP36L1, ZMIZ1, ZMYND8*

[a] This analysis only compared the SEs which occurred commonly in the two cell lines or tissues of each sample type, namely DDLPS cell lines, DDLPS tissues, MLPS cell lines, and MLPS tissues.

Supplementary Table 3: List of genes associated with FUS-DDIT3-defined super-enhancers in MLS402

Genes associated with FUS-DDIT3 defined SEs
AARS, AASDHPPT, ABCA1, ABCC2, ABCC4, ABCG1, ABHD5, ABI3BP, ABLIM3, ABTB2, AC002472.13, AC008394.1, AC008964.1, AC009403.2, AC011294.3, AC016251.1, AC022431.2, AC096677.1, AC108868.1, AC110084.1, AC112715.2, AC114494.1, AC117834.1, AC131097.4, ACKR3, ACTBL2, ACTL7B, ACTN1, ADAM12, ADAM17, ADAMTS12, ADAMTS14, ADAMTS16, ADAMTS6, ADAMTSL1, ADGB, ADORA2B, ADRB2, AF015262.2, AFAP1, AFF3, AGAP1, AGPAT4, AHR, AIM1, AJAP1, AK2, AKAP13, AKAP2, AKAP6, AKT3, AL035252.1, AL035610.2, AL133262.1, AL160175.1, AL162389.1, AL356215.1, ALCAM, ALDH1L1, ALDH8A1, ALOX5AP, ALPK2, AMOTL2, AMPD3, ANAPC1, ANKRD30B, ANKRD33B, ANKRD50, ANKRD55, ANO6, ANTXR2, ANXA1, ANXA10, ANXA2, ANXA2R, ANXA3, ANXA5, AP001055.1, AP2S1, AP5S1, APBB2, APCS, APOH, APPL2, ARAP2, AREGB, ARHGAP10, ARHGAP17, ARHGAP18, ARHGAP23, ARHGAP26, ARHGAP28, ARHGAP29, ARHGAP5, ARHGEF12, ARHGEF2, ARID5B, ARL15, ARL4C, ARNT, ARNTL, ARSJ, ARTN, ASAP1, ASAP2, ASB1, ASB2, ASB7, ASPH, ASTN1, ATF3, ATG7, ATOX1, ATP2B1, ATP2B2, ATP5E, ATP5O, ATP6V0C, ATP8A1, ATP9A, ATPIF1, ATXN1, AZIN1, B4GALT1, B4GALT5, BAAT, BACH1, BANP, BASP1, BBC3, BBS12, BCAS1, BCAT1, BCL11A, BCL2L10, BCL2L11, BDH1, BDKRB1, BDNF, BEND5, BHLHE40, BICC1, BLID, BLOC1S1, BMP4, BMP5, BMP7, BMPER, BNC1, BNC2, BOD1L1, BPI, BRD4, BRE, BRI3, BTD, C10orf76, C11orf74, C11orf87, C11orf88, C12orf79, C14orf132, C15orf53, C15orf54, C15orf61, C16orf72, C16orf97, C1GALT1, C1orf143, C1QTNF1, C20orf85, C21orf88, C2orf88, C2orf91, C3orf17, C3orf65, C5orf56, C5orf64, C6orf223, C7orf69, C8orf46, C9orf3, C9orf84, C9orf92, CA12, CAAP1, CACNA1C, CACNA2D3, CALM2, CALML6, CAMK2D, CAMTA1, CAP2, CAPN2, CARD18, CARHSP1, CARS, CASC17, CAST, CAV1, CAV2, CBX4, CCDC179, CCDC26, CCDC3, CCDC59, CCDC80, CCDC85B, CCL2, CCL20, CCR3, CCRN4L, CD164, CD200R1L, CD36, CD44, CDC42EP3, CDCA4, CDCP1, CDH13, CDH18, CDH2, CDH4, CDHR3, CDK6, CDKN2B, CDSN, CEBPB, CELSR1, CEP128, CEP152, CHD7, CHSY1, CIRBP, CITED2, CLDN16, CLEC14A, CLEC3B, CLIC4, CLIC5, CLIP1, CLIP2, CLIP4, CLMP, CNGB1, CNIH3, COA7, COL12A1, COL1A1, COL4A2, COL5A1, COL5A2, COL8A1, COLEC10, COMMD7, COPZ2, COQ2, CORO2B, COTL1, CPA3, CPA5, CPEB4, CPLX2, CREB1, CREB3L1, CRHBP, CRIM1, CRISPLD1, CRYL1, CRYZL1, CSMD3, CSNK1D, CSNK2A1, CST5, CT49, CTAGE1, CTD-2203A3.1, CTIF, CTLA4, CTPS1, CTSB, CTSC, CTSD, CUBN, CUEDC1, CXCL2, CXCL3, CXorf36, CYHR1, CYP1B1, CYP24A1, CYR61, DAAM1, DAB2, DAGLB, DAP, DCBLD1, DCBLD2, DCLK3, DCT, DDX10, DEC1, DEK, DENND2A, DGKD, DHRS2, DHX15, DIAPH3, DIO2, DKK1, DLC1, DLEU7, DLG1, DLGAP1, DLX2, DNAH5, DNAJB12, DNAJB6, DNAJC13, DNAJC7, DNASE2B, DNER, DPF3, DPP4, DPYD, DPYSL2, DPYSL3, DRAM1, DUSP1, DUSP10, DUSP5, DUSP6, DYNC2H1, DYNLRB2, DYSF, E2F7, EAPP, EBF1, ECI2, EDIL3, EDN1, EEF1E1, EFCAB1, EFEMP1, EFNA5, EGFLAM, EGFR, EGLN3, EHF, EIF2AK3, EIF2S2, EIF3F, ELFN2, ELMSAN1, ELTD1, EML1, EMP1, ENAH, ENO3, ENOX1, EPAS1, EPG5, EPHA2, EPHA3, EPHA5, EPM2A, ERBB2IP, ERC2, ERGIC1, ERI1, ERN1, ERRFI1, ESRRG, ETS1, ETS2, EVL, EXT1, EZH2, FAHD2A, FAM129A, FAM129B, FAM135B, FAM150B, FAM155A, FAM169B, FAM174B, FAM20C, FAM21A, FAM21C, FAM46A, FAM50B, FAT1, FBLIM1, FBLN1, FBN1, FBXL5, FEM1C, FGF9, FHAD1, FHL2, FILIP1, FILIP1L, FJX1, FKBP9, FLJ27365, FLNB, FLRT2, FMN1, FNDC3B, FOXD1, FOXN3, FOXP1, FRMD6, FST, FSTL5, FTH1, GADD45A, GADD45B, GADD45G, GADL1, GALNT12, GALNT15, GALNT2, GALNTL5, GALNTL6, GAP43, GARS, GAS7, GATA3, GBA, GBE1, GCLC, GCSH, GDF15, GET4, GFAP, GJA1, GLI3, GLRB, GLRX5, GLS, GMPR, GNA14, GNAL, GNAQ, GNLY, GOT2, GPHN, GPR125, GPR126, GPR88, GPRC5A, GPX1, GRAMD1B, GRID2, GRIK2, GRIN2A, GSE1, GSG1, GSK3B, GTF2IRD1, GTF2IRD2, GTF2IRD2B, GTPBP2, GUCY1A2, GYPC, HADH, HAS2, HDGFL1, HECW2, HERPUD1, HES1, HEY1, HIST2H2AA3, HIVEP2, HIVEP3, HLX, HM13, HMGA2, HMGCLL1, HNF4G, HNRNPF, HOXA3, HOXB9, HPCAL1, HS1BP3, HSF2BP, HSPA5, HSPA9, HTR1D, HUWE1, HYI, IER2, IER3, IFNE, IFT52, IGF1R, IGF2BP2, IGFBP3, IGSF23, IL1B, IL1RAP, IL1RL1, IL6, IL6ST, IL7, IL8, ILF3, INPP4B, IPO11, IQGAP1, IQGAP2, IRF1, IRF2BP2, IRF2BPL, IRG1, IRS1, IRS2, ITGA2, ITGB2, ITGB6, ITGBL1, IVNS1ABP, JADE1, JARID2, JPH3, KANK1, KANK4, KCNA4, KCNG1, KCNH7, KCNH8, KCNMA1, KCNS1, KCNU1, KCTD4, KDM6B, KIAA0430, KIAA0947, KIAA1324, KIAA1432, KIAA1549, KIF18A, KITLG, KLF2, KLF6, KLF7, KLHDC4, KLHL29, KLHL31, KLHL36, KLHL5, KLRC2, KRR1, KRT20, KRT81, KRTAP4-8, LACTB, LAMA2, LAMP1, LAPTM4A, LATS2, LHFPL2, LHX8, LIF, LIMD1, LIMS3, LINC00111, LINC00113, LINC00152, LINC00158, LINC00160, LINC00211, LINC00290, LINC00313, LINC00317, LINC00322, LINC00355, LINC00396, LINC00431, LINC00457, LINC00460, LINC00472, LINC00474, LINC00484, LINC00511, LINC00518, LINC00524, LINC00563, LINC00578, LINC00592, LINC00593, LINC00604, LINC00616, LINC00617, LINC00656, LINC00677, LINC00687, LINC00856, LINC00882, LINC00900, LINC00911, LINC00917, LINC00924, LINC00954, LINC00972, LINC00973, LINC01010, LINC01030, LINC01052, LINC01057, LINC01067, LINC01078, LINC01099, LINC01107, LINC01108, LIPC, LIPH, LITAF, LMX1A, LPAR1, LPIN1, LPIN2, LPP, LRIG1, LRIG3, LRRC16A, LRRC17, LRRC37A, LRRC37A3, LRRC49, LSAMP, LSM3, LSM6, LURAP1L, LYST, MACC1, MAF, MAFB, MAGEB2, MALAT1, MAMDC2, MAML2, MANBAL, MAP10, MAP3K5, MAP4K3, MAPK1, MAPK6, MAPRE1, MARCH1, MARCH10, MARCKS, MARK3, MAT2B, MATN2,

MAX, MB, MB21D2, MBD6, MBNL2, ME2, ME3, MED13L, MED15, MEG3, MERTK, MET, METTL15, MEX3B, MFSD1, MGAT4C, MIAT, MICAL2, MICAL3, MICALL1, MIR137HG, MIR146A, MIR181A1HG, MIR205HG, MIR29A, MIR378D2, MIR4458HG, MKLN1, MKNK2, MLPH, MMP10, MMP16, MMP17, MRPL36, MRPS16, MRVI1, MSH2, MSRB3, MTHFD1L, MTNR1B, MUSK, MYC, MYEOV, MYH3, MYH9, MYL12A, MYO10, MYO6, MYOF, NA, NABP1, NAMPT, NANOS3, NAV2, NBPF16, NCEH1, NCOR2, NDST1, NEAT1, NEDD1, NEDD4, NEDD9, NEGR1, NEK6, NEK7, NENF, NF2, NFATC2, NFE2L2, NFIB, NFIL3, NFKBIA, NFKBIZ, NGF, NHS, NKAIN2, NMBR, NMD3, NNMT, NNT, NOL8, NOTCH2, NOTCH2NL, NOVA1, NOX3, NPAS3, NPY, NR1D2, NRG1, NRXN1, NRXN3, NSMAF, NT5C1B, NT5E, NTM, NTMT1, NTN4, NTRK3, NUAK1, NUDT12, NXPE4, NYAP1, NYAP2, OCLN, ODF3B, OFCC1, OGFRL1, OR14K1, OR6J1, ORAOV1, OSGIN2, OSMR, OSR1, OTOL1, OXR1, OXSR1, PAM, PAPD7, PAQR5, PARD3B, PCDH1, PCGF5, PCID2, PCNX, PCSK1, PDE1C, PDE4D, PDE4DIP, PDGFC, PDLIM1, PEG10, PEPD, PFDN1, PGRMC2, PGS1, PHACTR1, PHC2, PHLDA1, PHLDA3, PHLDB2, PHOSPHO1, PHYKPL, PI4KB, PIEZO2, PIGL, PIK3C2G, PIK3CD, PIP4K2A, PISD, PKIG, PKP4, PLA2R1, PLAC1, PLAU, PLCXD2, PLD5, PLEC, PLEKHG3, PLET1, PLLP, PLOD2, PLS1, PLSCR5, PMEPA1, PMP22, PMS1, PNP, POC1A, PP13439, PPCDC, PPP1R12A, PPP1R15A, PPP1R15B, PPP1R3C, PPP2R3A, PREP, PREPL, PRIM2, PRIMPOL, PRKAG2, PRKCA, PRKCE, PRKCH, PRKD1, PRMT8, PRNP, PRR12, PRR15, PRR16, PSAP, PSAT1, PSCA, PSG4, PSG8, PSMB7, PSMD9, PTBP2, PTGER4, PTK2, PTPN1, PTPN12, PTPN14, PTPRC, PTPRD, PTPRK, PTPRQ, PVRL3, PXDN, PXN, PYDC2, QSOX1, RAB8B, RAD23B, RAD51B, RAG2, RAI14, RANBP17, RAP2B, RAPGEF2, RAPGEF4, RAPGEF5, RASGRF2, RASSF6, RBBP6, RBM39, RBM7, RBMS3, RBPJ, RCAN1, RCN1, REEP3, RERE, RFTN1, RGMB, RGS16, RGS20, RGS3, RGS5, RHBDF1, RHOBTB1, RIMS1, RIN3, RIPK4, RMND1, RND3, RNF145, RPL3, RPS6KA5, RRAS2, RRP15, RTN3, RTN4, RUNX2, RUSC2, RXFP2, S100PBP, SALL4, SAMD12, SAMD4A, SASH1, SATB1, SBF2, SBNO2, SBSPON, SCARB1, SCG2, SCHIP1, SCN8A, SDC3, SDC4, SDK2, SDPR, SEMA3A, SEMA3C, SEMA5A, SERP2, SERPINA3, SERPINB2, SERPINB8, SERPINE1, SETD5, SETD7, SETD8, SEZ6L, SGIP1, SH2B3, SH3BP4, SH3RF1, SH3RF2, SH3TC2, SHB, SIGLEC15, SIM1, SIPA1L1, SIPA1L2, SLA, SLAIN2, SLC12A8, SLC16A5, SLC29A3, SLC2A1, SLC35F6, SLC38A1, SLC38A2, SLC45A1, SLC4A3, SLC4A7, SLC7A11, SLC7A14, SLC7A5, SLC8A1, SLC9A1, SLC9A4, SLFN5, SLIT2, SLIT3, SLX4IP, SMAD3, SMC2, SMNDC1, SMOX, SMS, SMURF2, SMYD3, SNAP25, snoU13, SNTB1, SNX13, SNX19, SNX29P2, SOCS3, SOCS5, SOD3, SOGA2, SOWAHB, SOX11, SP3, SPATA32, SPCS3, SPG20, SPIRE1, SPOCD1, SPOPL, SPP2, SPRY4, SPTBN1, SRGAP1, SRGN, ST3GAL3, STARD13, STC2, STEAP1B, STK4, STK40, SUCNR1, SUGCT, SUPT3H, SUPV3L1, SUSD1, SYBU, SYN3, SYNPO, SYT16, TANC2, TAS2R1, TBC1D23, TBX18, TBX3, TCEAL1, TCF7L2, TDRD15, TEAD1, TENM2, TENM3, TES, TEX41, TFAP2A, TFAP2C, TGFB2, TGFBI, TGFBR2, TGM2, TGS1, THBS1, THBS2, THSD4, TIMP3, TIMP4, TINAG, TLK1, TLL1, TMCO5A, TMEM107, TMEM14E, TMEM19, TMEM194A, TMEM196, TMEM2, TMEM212, TMEM41B, TMEM44, TMEM52B, TMEM72, TMEM75, TMTC3, TNFRSF10B, TNFRSF1A, TNFSF18, TNFSF4, TNIK, TNIP1, TOMM20, TOP1, TOX2, TP53TG3, TPCN1, TPCN2, TPM1, TRABD2A, TRDMT1, TRIB1, TRIB3, TRIM25, TRIM37, TRIM43, TRIM55, TRIO, TRIOBP, TRPC4, TRPC6, TSC22D2, TSEN15, TSN, TSPAN14, TUB, TUFT1, TULP3, TUSC1, TXNDC2, TXNRD1, U91319.1, UACA, UAP1, UBAP2, UBASH3B, UBC, UBE2E1, UBE2G1, UBE2Q1, UBE2QL1, UGCG, UPP1, UQCC1, UQCRC1, USP24, USP25, USP3, UST, UTRN, VANGL1, VAT1L, VEGFC, VEPH1, VMP1, VPS53, VPS72, WDFY3, WDR25, WDR27, WDR66, WDR70, WDR75, WDR81, WISP2, WWC1, WWTR1, XIRP2, XPNPEP1, XPOT, YTHDF3, ZBTB1, ZBTB12, ZBTB38, ZCCHC17, ZFAND3, ZFAT, ZFHX4, ZFP36L1, ZFP36L2, ZFP64, ZMIZ1, ZMYM1, ZMYND8, ZNF217, ZNF358, ZNF365, ZNF385B, ZNF385D, ZNF469, ZNF706, ZNF710, ZNF827, ZSWIM6

Supplementary Table 4: Oligoes used for shRNA, sgRNA cloning and qPCR analysis

shRNA	Sequence of forward primer (5' -> 3')
sh-MYC-1	CCGGCCTGAGACAGATCAGCAACAACTCGAGTTGTTGCTGATCTGTCTCAGGTTTTTG
sh-MYC-2	CCGGCCCAAGGTAGTTATCCTTAAACTCGAGTTTAAGGATAACTACCTTGGGTTTTTG
sh-FOSL2-1	CCGGGCAGAAATTCCGGGTAGATATCTCGAGATATCTACCCGGAATTTCTGCTTTTTG
sh-FOSL2-2	CCGGGTGATCACCTCCATGTCCAATCTCGAGATTGGACATGGAGGTGATCACTTTTTG
sh-RUNX1-1	CCGGTCGCCCTGTTTGGCATCTAATCTCGAGATTAGATGCCAAACAGGGCGATTTTTG
sh-RUNX1-2	CCGGGAACCAGGTTGCAAGATTTAACTCGAGTTAAATCTTGCAACCTGGTTCTTTTTG
sh-CBFB-1	CCGGTGACCTCAAACTTCGTTAATTCTCGAGAATTAACGAAGTTTGAGGTCATTTTTG
sh-CBFB-2	CCGGGAGAAGCAGGCAAGGTATATTCTCGAGAATATACCTTGCCTGCTTCTCTTTTTG
sh-SNAI2-1	CCGGGAGTGACGCAATCAATGTTTACTCGAGTAAACATTGATTGCGTCACTCTTTTTG
sh-SNAI2-2	CCGGCCGAAGCCAAATGACAAATAACTCGAGTTATTTGTCATTTGGCTTCGGTTTTTG
sh-BRD2-1	CCGGCCTATGGACATGGGTACTATTCTCGAGAATAGTACCCATGTCCATAGGTTTTTG
sh-BRD2-2	CCGGCTACCACTGTCCTCAACATTCCTCGAGGAATGTTGAGGACAGTGGTAGTTTTTG
sh-BRD3-1	CCGGGTGAGATTCGTACCGAAGAACCTCGAGGTTCTTCGGTACGAATCTCACTTTTTG
sh-BRD3-2	CCGGCAAATTGAACCTGCCGGATTACTCGAGTAATCCGGCAGGTTCAATTTGTTTTTG
sh-BRD4-1	CCGGGCCAAATGTCTACACAGTATACTCGAGTATACTGTGTAGACATTTGGCTTTTTG
sh-BRD4-2	CCGGTGAACCTCCCTGATTACTATACTCGAGTATAGTAATCAGGGAGGTTCATTTTTG
sh-CRBN	CCGGGCCCACGAATAGTTGTCATTTCTCGAGAAATGACAACTATTCGTGGGCTTTTTG
sh-DDB1	CCGGTCCACTAGATCGCGATAATAACTCGAGTTATTATCGCGATCTAGTGGATTTTTG
sh-RBX1	CCGGTGGGATATTGTGGTTGATAACCTCGAGGTTATCAACCACAATATCCCATTTTTG
sh-DDIT3	CCGGGCCAATGATGTGACCCTCAATCTCGAGATTGAGGGTCACATCATTGGCTTTTTG
**sgRNA**	**Sequence of forward primer (5' -> 3')**
sg-BRD2-1	CACCGACCACTCTCTCTACGCATAG
sg-BRD2-2	CACCGATAAAACAGCCTATGGACAT
sg-BRD3-1	CACCGCATCACTGCAAACGTCACGT
sg-BRD3-2	CACCGCACAACCACTCCCACGACGT
sg-BRD4-1	CACCGAGTCGAACTGTCACTGTCCG
sg-BRD4-2	CACCGAGTCGATTTCAATCTCGTCG
**qPCR**	**Sequence (5' -> 3')**
ACTB-F	GACGACATGGAGAAAATCTG
ACTB-R	ATGATCTGGGTCATCTTCTC
AIM1-F	GACAGTGACCACTAAAGTGACC
AIM1-R	GTGGCAGTGTTGCCTTTGT
ARID5B-F	CAGAAGAATGCTGAGCCAAC
ARID5B-R	TGGGAAACTATTGGCACGTA
ATF3-F	CCTCTGCGCTGGAATCAGTC
ATF3-R	TTCTTTCTCGTCGCCTCTTTTT
BCAT1-F	GAGCCTGGAAAGGTGGAACTG
BCAT1-R	GCTGACACCCATTATCTACTGCT
CDKN1A-F	CCTGTCACTGTCTTGTACCCT
CDKN1A-R	GCGTTTGGAGTGGTAGAAATCT
CRADD-F	CATCAGACCGGCAGATTAACC
CRADD-R	GTTGGCCTTACAGCGGTAGAT
DDX10-F	GAAGCTCAGTACCGTTTGGTG
DDX10-R	CTGATAGGCCAGTTCTCTCGTA
FOSL2-F	AAGAAAAACACCCTGTTTCC
FOSL2-R	TATCTACCCGGAATTTCTGC
FST-F	AGGCAAGATGTAAAGAGCAGC
FST-R	CAGTAGGCATTATTGGTCTGGTC
GAPDH-F	GTCAGTGGTGGACCTGACCT
GAPDH-R	AGGGGTCTACATGGCAACTG
HMGA2-F	ACCCAGGGGAAGACCCAAA
HMGA2-R	CCTCTTGGCCGTTTTTCTCCA
IL8-F	ACTGAGAGTGATTGAGAGTGGAC
IL8-R	AACCCTCTGCACCCAGTTTTC

KANK1-F	AGTACAGCTTAGAGAAACCACCC
KANK1-R	TGTCAACCTTTTTCCTCGATCC
MYOF-F	ATCCGCGTCCGAGTGATTG
MYOF-R	ACAGACGTGAACTTTGACCAC
MYC-F	TGAGGAGGAACAAGAAGATG
MYC-R	ATCCAGACTCTGACCTTTTG
POLA1-F	AAAGATCCATTGGAGCTTCACC
POLA1-R	TCAGCACGTTTAAGAGGAACAG
PPARG-F	TCTCTCCGTAATGGAAGACC
PPARG-R	GCATTATGAGACATCCCCAC
RUNX1-F	TTGTGATGCGTATCCCCGTAGA
RUNX1-R	GCCGAGTAGTTTTCATCATTGCC
SAT1-F	ACCCGTGGATTGGCAAGTTAT
SAT1-R	TGCAACCTGGCTTAGATTCTTC
SMURF2-F	TATGCAAACTCGGGCCAAATG
SMURF2-R	CCTGTGCCTATTCGGTCTCTG
SNAI2-F	TGCATATTCGGACCCACACATTA
SNAI2-R	ATTTGACCTGTCTGCAAATGCTC
SOCS1-F	CACGCACTTCCGCACATTC
SOCS1-R	TAAGGGCGAAAAAGCAGTTCC

Supplementary Table 5: List of key chemicals

Chemicals	Company
(+)-JQ1	Tocris
I-BET151	Sigma-Aldrich
OTX015	Selleckchem
CPI203	ApexBio
dBET1	Chemietek
dBET6	MedChemExpress
ARV-825	Chemietek
Cycloheximide	Sigma-Aldrich
MG132	Sigma-Aldrich
Kolliphor® HS 15	Sigma-Aldrich
Gemcitabine , Hydrochloride Salt	LC Laboratories
Docetaxel	LC Laboratories
Paclitaxel	LC Laboratories

Supplementary Table 6: List of antibodies

Antibody	Company	Catalog	Application
α-Tubulin	Santa Cruz	sc-8035	WB
β-ACTIN	Sigma-Aldrich	A1978	WB
BRD1	Santa Cruz	sc-398226	WB
BRD2	Proteintech	22236-1-AP	WB, ChIP-seq
BRD3	Proteintech	11859-1-AP	WB
BRD3	Bethyl	A302-367A	ChIP-seq
BRD4	Cell Signaling Technology	13440	WB
BRD4	Bethyl	A301-985A	WB, IP, ChIP-seq
CBFB	Cell Signaling Technology	62184	WB
CRBN	Sigma-Aldrich	SAB1407456	WB
CRBN	Sigma-Aldrich	HPA045910	WB
DDB1	GeneTex	GTX100130	WB
DDIT3	Cell Signaling Technology	2895	WB, IP, ChIP-seq
FLAG	Sigma-Aldrich	F1804	WB
FOSL2	Santa Cruz	sc-604X	ChIP-seq
FOSL2	Cell Signaling Technology	19967	WB
GAPDH	Cell Signaling Technology	2118	WB
GFP	MBL	598	WB
H3K27ac	Activemotif	39133	ChIP-seq
H3K27ac	Abcam	ab4729	ChIP-seq
H3K4me3	Emdmillipore	04-745	ChIP-seq
H3K4me1	Abcam	ab8895	ChIP-seq
Histone H3	Cell Signaling Technology	4499	WB
MYC	Cell Signaling Technology	5605	WB
p21	Cell Signaling Technology	2947	WB
Pan-RUNX	Abcam	ab92336	WB, IP, ChIP-seq
RBX1	Santa Cruz	sc-393640	WB
RNA-Pol2	Santa Cruz	sc-899X	ChIP-seq
RUNX1	Santa Cruz	sc-365644	WB
SNAI2	Cell Signaling Technology	9585	WB

Supplementary Figure 1. Relative ranks of stitched enhancer domains.

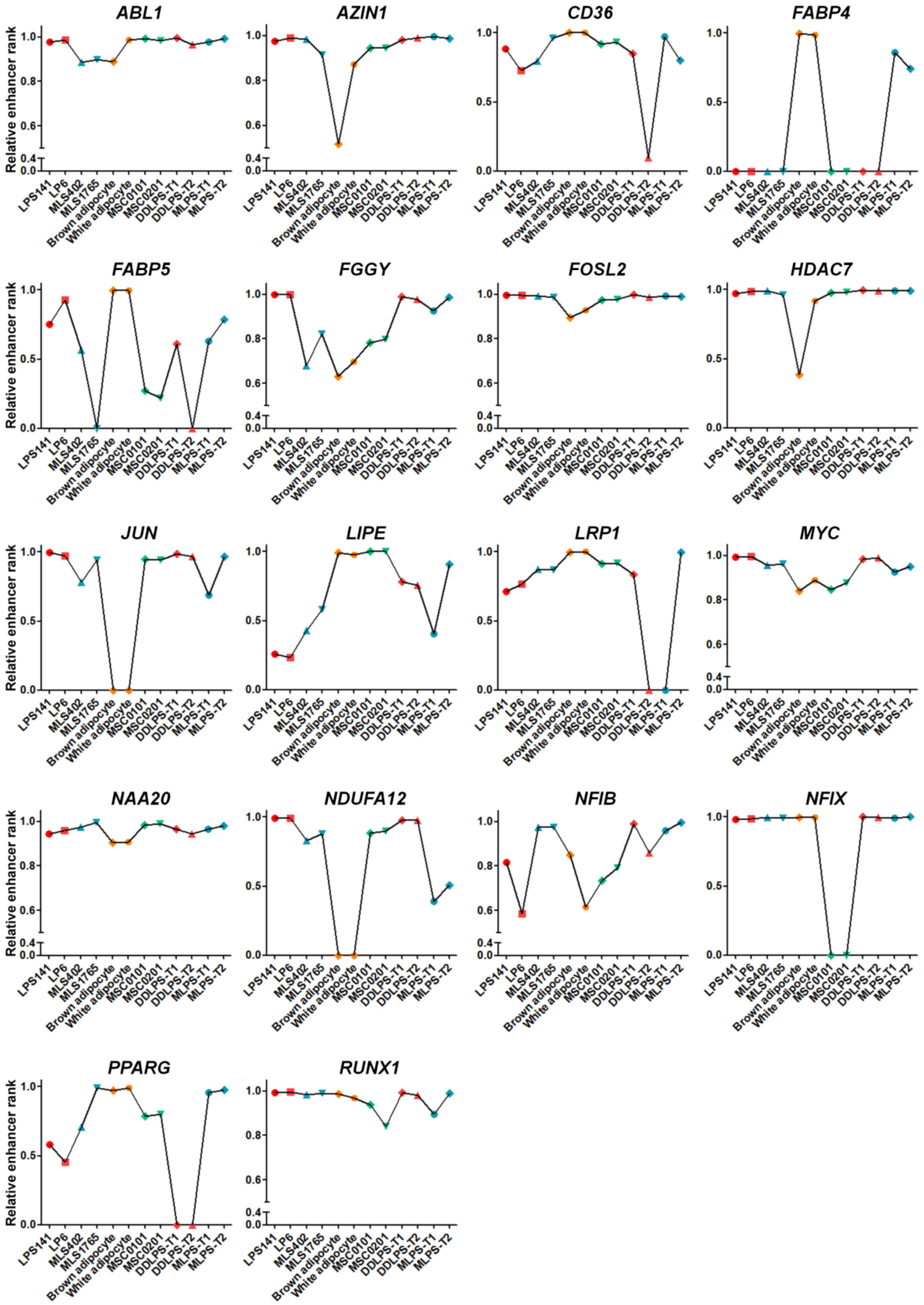

Each line chart showed relative ranks of stitched enhancer domains that were associated with indicated genes among cell lines and tissue samples.

Supplementary Figure 2. Expression and enrichment analyses of super-enhancer-associated genes.

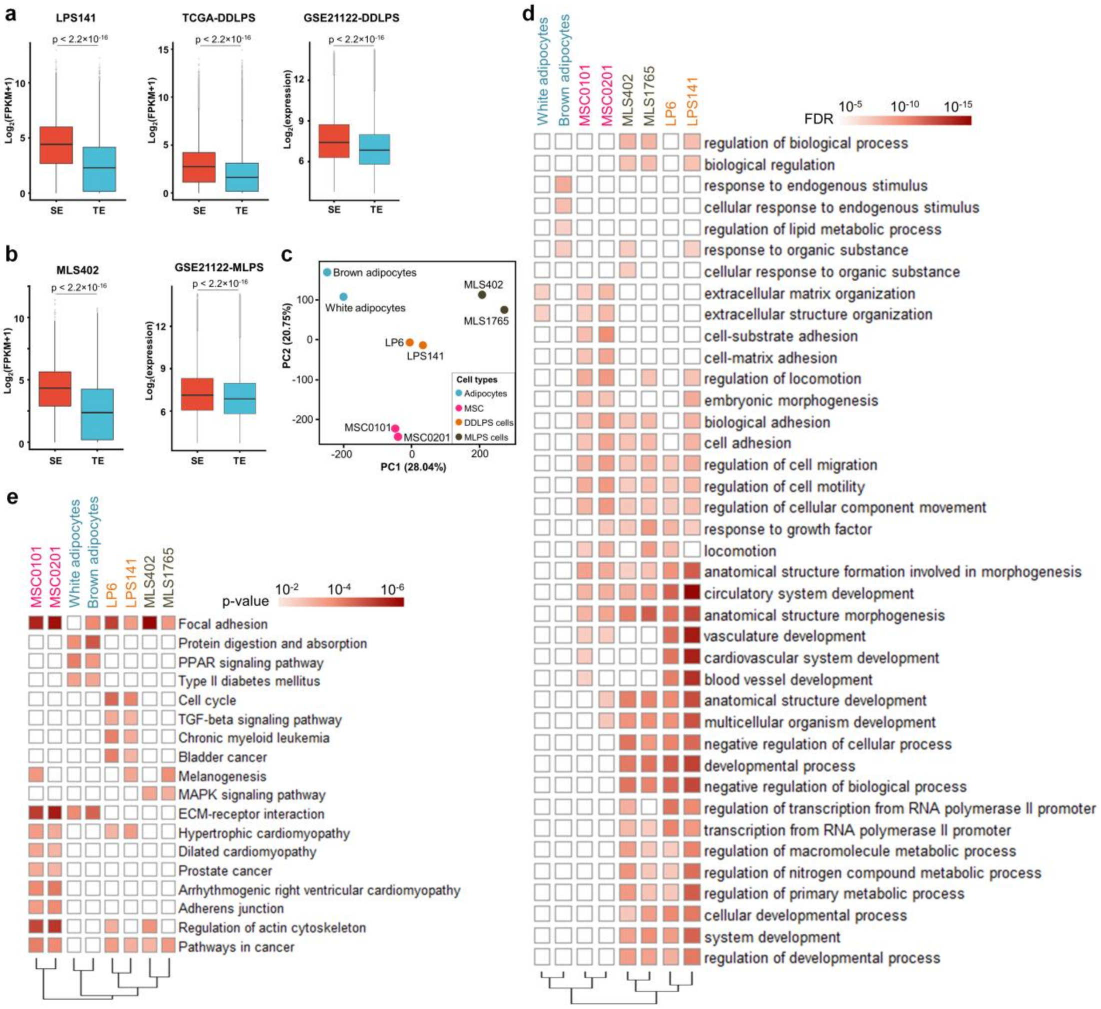

a,**b**, SE-associated genes in LPS cells show higher expression levels than TE-associated genes in cell lines and primary samples of (**a**) DDLPS and (**b**) MLPS. SE, super-enhancer; TE, typical enhancer. Box plots indicate median value (center line), first and third quartiles (box limits), as well as minimum and maximum values (whiskers) after excluding outliers (dots). Wilcoxon signed-rank test was applied. **c**, Principal component (PC) analysis plot showing the variation of SE structures among 8 cell line samples. **d**, Heat map displaying enrichment of SE-associated genes in various gene ontology (GO) terms. Top 10 GO terms in each sample with false discovery rate (FDR) cutoff of 0.0001 are shown. Any value above 0.0001 was converted to 1 for heat map illustration. **e**, Heat map demonstrating enrichment of SE-associated genes in Kyoto Encyclopedia of Genes and Genomes (KEGG) pathways. KEGG pathways that were significantly overrepresented in at least two samples are shown. Hypergeometric test was applied. Any p-value above 0.01 was converted to 1 for heat map illustration. Source data are provided as a Source Data file.

Supplementary Figure 3. Expression and function of FUS-DDIT3 in MLPS.

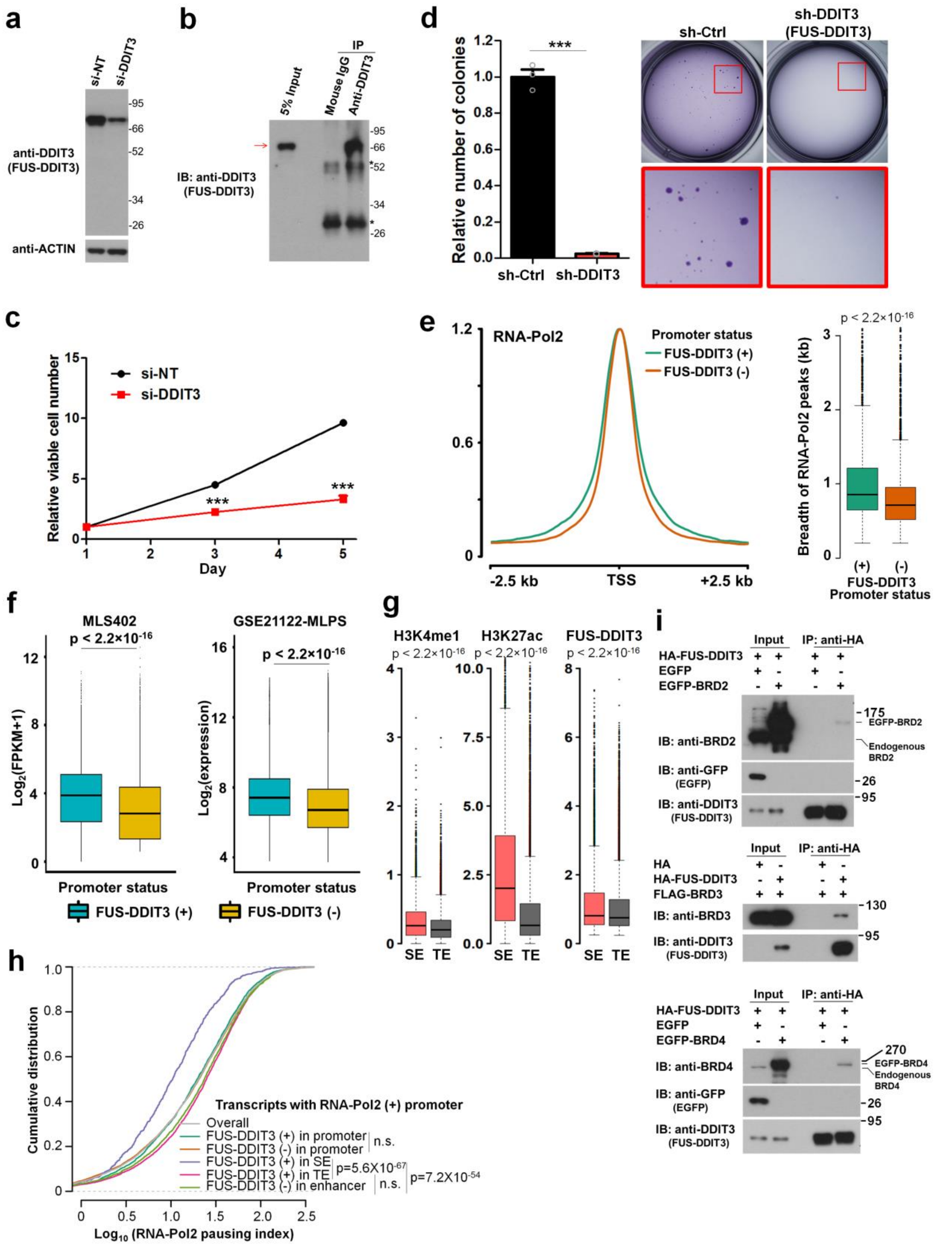

a, Immunoblot analysis displaying prominent expression of FUS-DDIT3 in MLS402 cells. Endogenous wild-type DDIT3 (about 27 kDa) was undetectable in this analysis. MLS402 cells transfected with si-DDIT3 showed a marked reduction in FUS-DDIT3 protein. **b**, Immunoprecipitation of FUS-DDIT3 by anti-DDIT3 antibody in MLS402 cells. Arrow indicates the FUS-DDIT3 band. Stars mark immunoglobulin heavy and light chains. **c**,**d**, Effects of DDIT3 silencing on (**c**) cell viability, and (**d**) anchorage-independent growth of MLS402 cells. Data are presented as mean ± SEM; *n* = 3. Two-tailed

Student's t-test was used. **e**, Differential loading of RNA-Pol2 in promoters either with or without FUS-DDIT3 occupancy. Wilcoxon rank sum test was applied for the statistical analysis. **f**, Genes with their promoters bound by FUS-DDIT3 in MLS402 cells demonstrate higher expression levels in MLS402 cells and primary MLPS samples. Wilcoxon signed-rank test was applied. **g**, Differential enrichment of FUS-DDIT3 in SE and TE regions. Y axis represents the average peak height (rpm). Welch two sample t-test was applied. **h**, RNA-Pol2 pausing index at epigenetically defined gene sets in MLPS cells. All transcripts with RNA-Pol2 binding signals at their promoters (n = 12,319) were included for calculation of RNA-Pol2 pausing index. Subcategory gene sets were defined based on the FUS-DDIT3 binding status at either promoter or stitched enhancer. Pairwise Wilcoxon test was applied. **i**, Co-IP assay showing the interaction between FUS-DDIT3 and BET proteins. HA-FUS-DDIT3 was co-expressed with EGFP-BRD2, EGFP-BRD4 or FLAG-BRD3 in HEK293T cells. Box plots indicate median value (center line), first and third quartiles (box limits), as well as minimum and maximum values (whiskers) after excluding outliers (dots). Source data are provided as a Source Data file.

Supplementary Figure 4. Identification of core transcriptional regulatory circuitry in DDLPS.

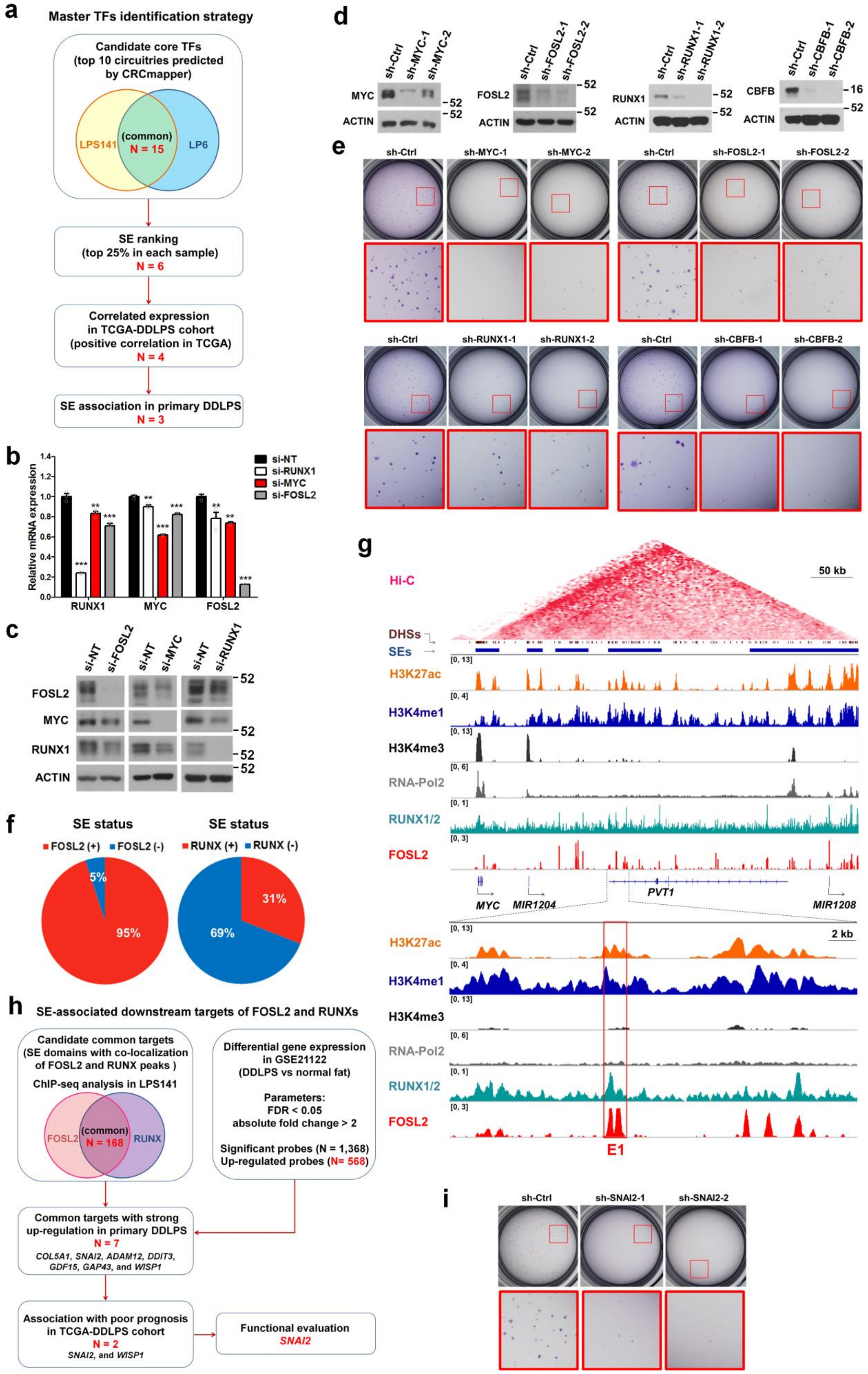

a, Workflow describing SE associated core TFs prioritization for experimental and functional evaluation. **b**,**c**, Effects of core TF knockdown on the (**b**) mRNA and (**c**) expression of indicated TFs. RNA was extracted from LPS141 cells 36 hours after siRNA transfection and subjected to qRT-PCR analysis. Data are presented as mean ± SEM; n = 3. Significance was reported within each target gene based on one-way ANOVA. Whole cell lysate was extracted from LPS141 cells 72 hours after siRNA transfection and subjected to immunoblot analysis. **d**,**e**, Effects of shRNA-mediated silencing of core TFs and CBFB on (**d**) respective protein expression and (**e**) anchorage-independent growth of LPS141 cells. **f**, Pie charts showing enrichment of RUNX and FOSL2 occupancy in SE regions. **g**, Co-occupancy of RUNX and FOSL2 across the *MYC* SEs, including a known *MYC* enhancer (E1) inside the intron 1 of *PVT1* locus (Fulco et al., 2016). Hi-C data from IMR90 were used to indicate the long-range chromatin interactions (query via http://promoter.bx.psu.edu/hi-c). DNase I hypersensitive sites (DHSs) were available from the ENCODE (query via http://promoter.bx.psu.edu/hi-c). **h**, Workflow describing the selection criteria and hits of SE-associated downstream targets of FOSL2 and RUNX proteins in DDLPS. **i**, Effect of SNAI2 knockdown on soft agar colony forming ability of LPS141 cells. Source data are provided as a Source Data file.

Supplementary Figure 5. Expression and function of BET genes in LPS.

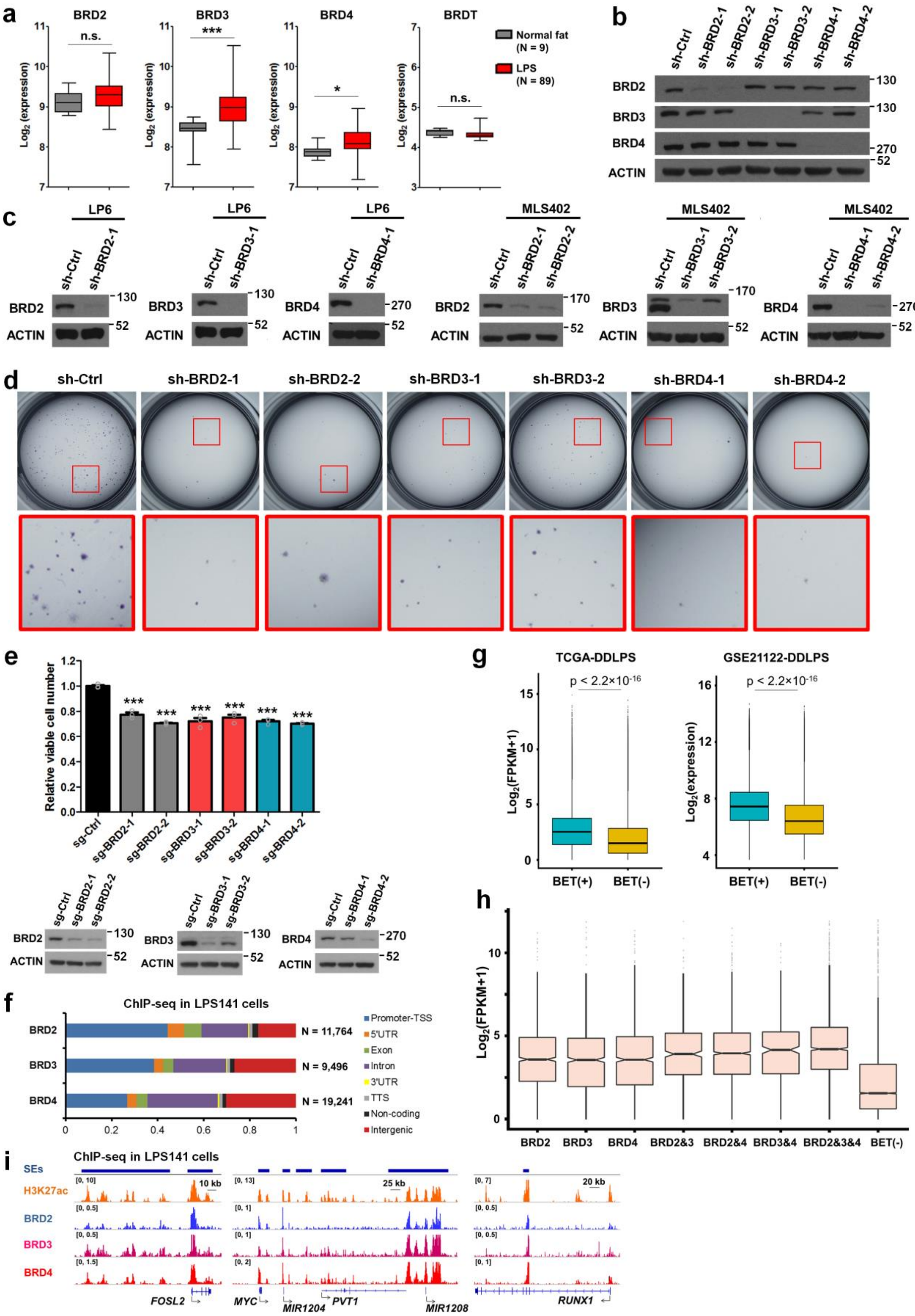

a, Comparison of BRD2, BRD3, BRD4, and BRDT transcripts in LPS samples and normal fat tissues (Normal). BRDT transcript was relatively less abundant and remained unchanged in LPS. Two-tailed Student's t-test was applied. cDNA microarray data were retrieved from GSE21122. **b**,**c**, Immunoblot analysis showing knockdown efficiency of shRNAs targeting BET genes. **d**,

Effect of BET gene knockdown on soft agar colony forming ability of LPS141 cells. **e**, Effect of CRISPR/Cas9-mediated silencing of BET genes on target protein expression and viability of LPS141 cells. One-way ANOVA was applied. **f**, Peak distribution of BET proteins across the LPS141 genome. **g**, Differential expression of BET (+) genes and BET (-) genes in two independent cohorts of DDLPS patients. Wilcoxon signed-rank test was applied. **h**, Differential levels of basal expression of BET targets and BET (-) genes in LPS141 cells. **i**, Co-occupancy of BRD2, BRD3, and BRD4 proteins across the SE regions of core TF genes in LPS141cells. Box plots indicate median value (center line), first and third quartiles (box limits), as well as minimum and maximum values (whiskers) after excluding outliers (dots). Source data are provided as a Source Data file.

Supplementary Figure 6. CRBN-dependent activities of ARV-825.

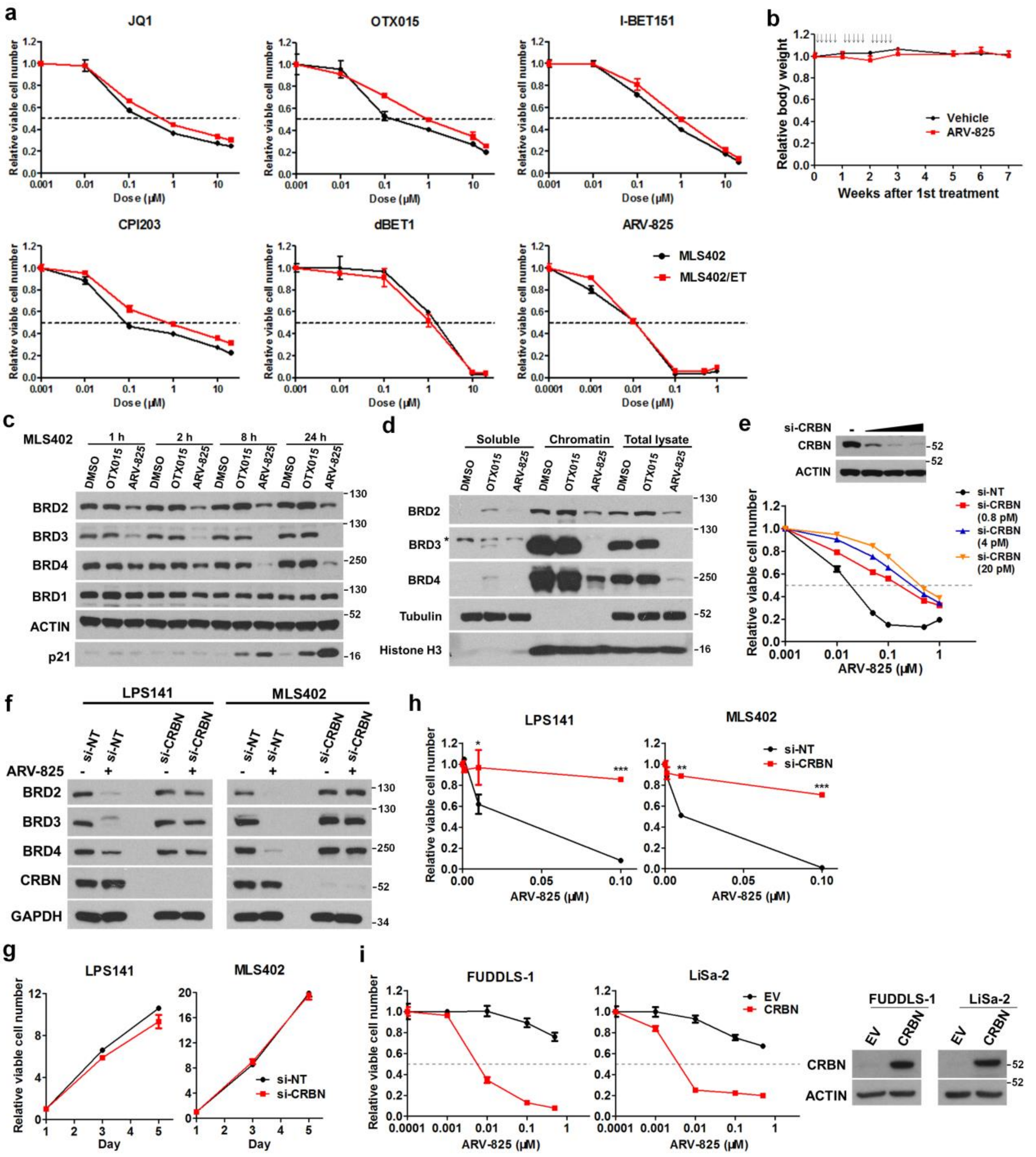

a, Dose-effect curves showing responses of MLS402 cells and their Trabectedin-resistant isogenic MLS402/ET counterparts to iBETs and dBETs. MTT assay was used to determine cell viability 96-hour post treatment ($n \geq 2$). **b**, Effect of ARV-825 treatment on body weight of recipient mice ($n = 4$) bearing MLS402 xenograft. **c**, Temporal effects of ARV-825 (200 nM) and OTX015 (200 nM) on the protein levels of BRD1/2/3/4 and p21 in MLS402 cells. **d**, Effects of equimolar ARV-825 and OTX015 (200 nM, 24 hours) on levels of chromatin-bound and non-bound fractions of BET proteins in LPS141 cells. Tubulin and Histone H3 were used as loading control for soluble and chromatin fraction, respectively. **e**, Effect of CRBN silencing on the anti-proliferative efficacy of ARV-825. LPS141 cells were transfected with either si-NT or indicated amount of si-CRBN and subjected to ARV-825 treatment.

MTT assay was used to determine cell viability 96-hour post treatment. **f**-**h**, Effects of siRNA-mediated depletion of CRBN on (**f**) ability of ARV-825 to deplete BET proteins, (**g**) cell viability, and (**h**) anti-proliferative activity of ARV-825. Two-tailed Student's t-test was used to compare the statistical difference between two groups (**h**) at each dose. **i**, Effect of CRBN overexpression on responsiveness of LPS cells to ARV-825 treatment. Data of (**a**, **b**, **e**, and **g**-**i**) are presented as mean ± SEM; $n \geq 2$. Source data are provided as a Source Data file.

Supplementary Figure 7. Effects of BET targeting agents and chemotherapy drugs on the gene expression in LPS cells.

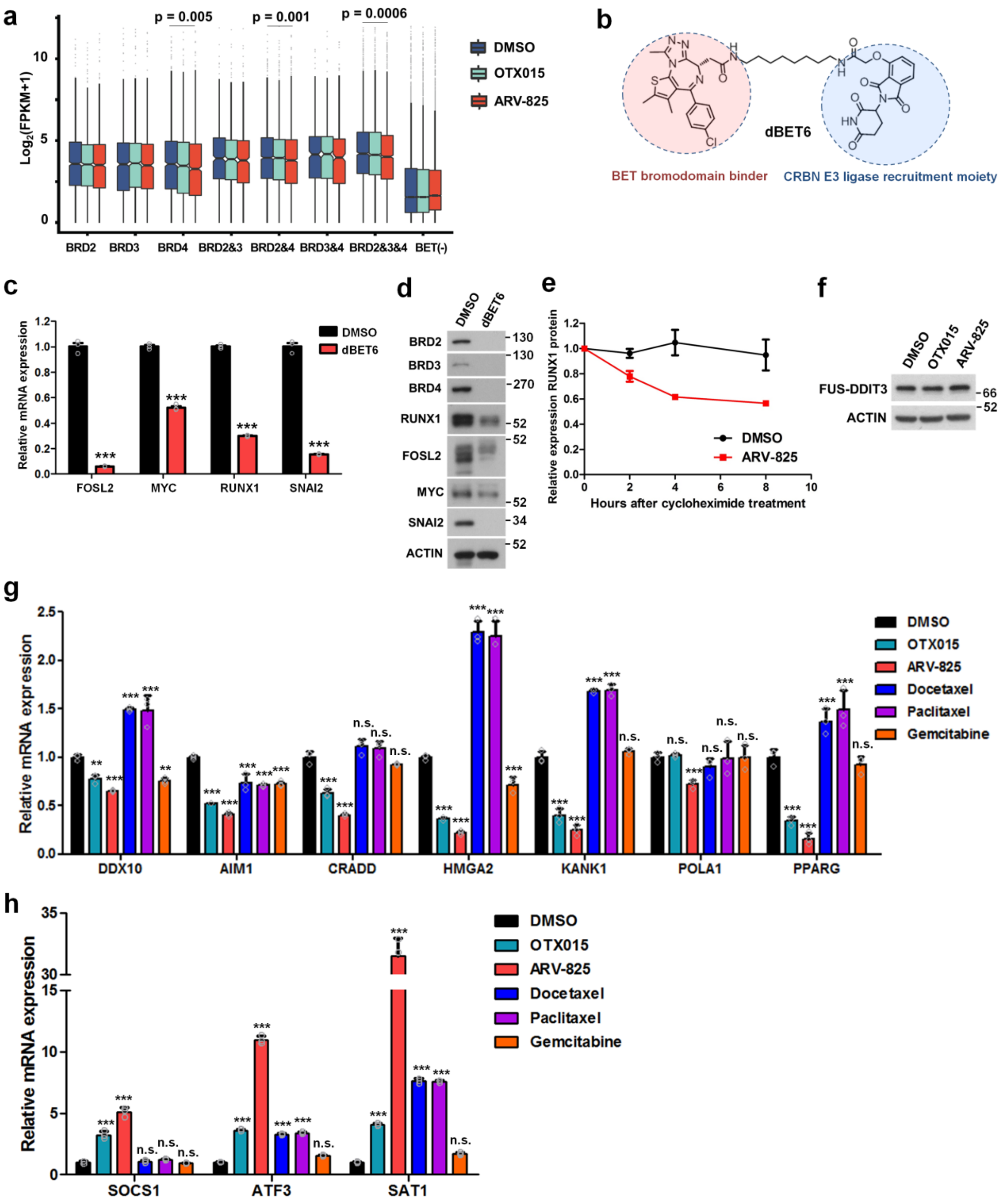

a, Differential responses of BET targets and BET (-) genes to ARV-825 and OTX015 treatment in LPS141 cells. LPS141 cells were treated with either OTX015 or ARV-825 (200 nM, 24 hours) before RNA extraction. Each category represents a group of genes with their promoter-proximal regions bound by indicated BET proteins. BET(-) denotes genes that are negative for binding of BET proteins. Box plot indicates median value (center line), first and third quartiles (box limits), as well as minimum and maximum values (whiskers) after excluding outliers (dots). Significance was reported within each category based on Wilcoxon signed-rank test. **b**, Chemical structure of dBET6. **c**, Effect of dBET6 treatment (100 nM, 24 hours) on the mRNA levels

of FOSL2, MYC, RUNX1, and SNAI2 in LPS141 cells. Two-tailed Student's t-test was used. ****p* < 0.001. **d**, Effect of dBET6 treatment (100 nM, 24 hours) on the expression of BET proteins, core TFs, and SNAI2 in LPS141 cells. **e**, Effect of ARV-825 treatment on the expressional level of RUNX1 protein. LPS141 cells were treated with either DMSO or ARV-825 (200 nM, 24 h) and incubated with cycloheximide (100 µg/mL) for indicated durations. Immunoblotting results were quantified by ImageJ and normalized to ACTIN. Relative expression of RUNX1 was calculated by comparing RUNX1 intensity at each time point after normalization to Time 0. **f**, Immunoblot analysis showing the impact of ARV-825 and OTX015 (200 nM, 24 hours) on protein level of FUS-DDIT3 in MLS402 cells. **g**,**h**, Differential effects of BET targeting agents and chemotherapy drugs on the expression of the signature genes that are known to be either (**g**) downregulated or (**h**) upregulated by Trabectedin. MLS402 cells were treated with OTX015 (200 nM, 24 hours), ARV-825 (200 nM, 24 hours), docetaxel (10 nM, 24 hours), paclitaxel (10 nM, 24 hours), and gemcitabine (50 nM, 24 hours) before RNA extraction. Data are presented as mean ± SEM. Statistical results from one-way ANOVA were reported. Source data are provided as a Source Data file.
