# Supplementary material for: Bromodomain and extraterminal proteins foster the core transcriptional regulatory programs and confer vulnerability in liposarcoma
Source: Nat Commun. 2019 Mar 22;10:1353. doi: 10.1038/s41467-019-09257-z (PMC6430783; doi:10.1038/s41467-019-09257-z)
Supplement: Supplementary file 2 — Reporting Summary [file 41467_2019_9257_MOESM3_ESM.pdf]

natureresearch

Corresponding author(s): Liang Xu

# Reporting Summary

Nature Research wishes to improve the reproducibility of the work that we publish. This form provides structure for consistency and transparency in reporting. For further information on Nature Research policies, see Authors & Referees and the Editorial Policy Checklist.

## Statistical parameters

When statistical analyses are reported, confirm that the following items are present in the relevant location (e.g. figure legend, table legend, main text, or Methods section).

| n/a | Confirmed | |
|---|---|---|
| ☐ | ☒ | The exact sample size (n) for each experimental group/condition, given as a discrete number and unit of measurement |
| ☐ | ☒ | An indication of whether measurements were taken from distinct samples or whether the same sample was measured repeatedly |
| ☐ | ☒ | The statistical test(s) used AND whether they are one- or two-sided<br>*Only common tests should be described solely by name; describe more complex techniques in the Methods section.* |
| ☒ | ☐ | A description of all covariates tested |
| ☐ | ☒ | A description of any assumptions or corrections, such as tests of normality and adjustment for multiple comparisons |
| ☐ | ☒ | A full description of the statistics including central tendency (e.g. means) or other basic estimates (e.g. regression coefficient) AND variation (e.g. standard deviation) or associated estimates of uncertainty (e.g. confidence intervals) |
| ☐ | ☒ | For null hypothesis testing, the test statistic (e.g. *F*, *t*, *r*) with confidence intervals, effect sizes, degrees of freedom and *P* value noted<br>*Give P values as exact values whenever suitable.* |
| ☒ | ☐ | For Bayesian analysis, information on the choice of priors and Markov chain Monte Carlo settings |
| ☒ | ☐ | For hierarchical and complex designs, identification of the appropriate level for tests and full reporting of outcomes |
| ☒ | ☐ | Estimates of effect sizes (e.g. Cohen's *d*, Pearson's *r*), indicating how they were calculated |
| ☐ | ☒ | Clearly defined error bars<br>*State explicitly what error bars represent (e.g. SD, SE, CI)* |

*Our web collection on statistics for biologists may be useful.*

## Software and code

Policy information about availability of computer code

| | |
|---|---|
| Data collection | ChIP-seq data of adipocytes (GSE59703; sequence length = 50) and mesenchymal stem cells (GSE16256; sequence length = 36) were downloaded from GEO. Transcriptome data for DDLPS samples of Sarcoma cohort (TCGA-SARC, N = 57) were downloaded using TCGABiolinks Bioconductor package via GDC (data freeze from Dec-2017). Gene Expression Matrix files for microarray dataset GSE21122 were downloaded using GEOquery Bioconductor package. |
| Data analysis | For ChIP-seq data analysis, raw reads were aligned to hg19 reference genome using bowtie aligner followed by removal of PCR duplicates with Picard markDuplicates utility. Resulting bam files were used for peak calling with MACS2 by extending reads to 200 bp. ChIP signals (Bedgraph) were simultaneously generated and input signal was subtracted with MACS2 bdgmp function. Bedgraphs were later converted to bigwig files with UCSC bedGraphTobigWig utility. Alignment and peak calling pipelines with hard-coded parameters are available at https://github.com/PoisonAlien/chiptk. Detected peaks were annotated with HOMER annotatePeaks. De novo motif identification and comparison were also performed with HOMER using findMotifsGenome program. Heatmaps were drawn using deeptools by centering either on identified peaks or on Transcription Start Site (TSS) of known refseq genes. For profile plots, signals around +/- 2,500 bp of peak center were extracted for every 25 bp bin using bwtool matrix function. Average signal was estimated and plotted in R. ChIP-seq data of adipocytes (GSE59703; sequence length = 50) and mesenchymal stem cells (GSE16256; sequence length = 36) have been published previously.<br>The ROSE (Rank Ordering of Super-Enhancers) algorithm (https://bitbucket.org/young_computation/rose) was used to call super-enhancers (SEs). Regions within +/- 1,250 bp from TSS were excluded for this analysis. Input-subtracted ChIP-seq signals were stitched and ranked based on intensity. A geometrical inflection point was used as cut-off to separate SEs from typical enhancers. SE-association was annotated with Ensemble genes. For PCA and hierarchical clustering analysis, ChIP signals for all SEs identified across samples were |

estimated (area under the peak) followed by log2 transformation. PCA was performed with prcomp function. Same data were used for Hierarchical clustering with hclust function. Core transcriptional regulatory circuitries were computationally inferred by CRCmapper based on scanning of TF motifs within SEs.
The peak distribution of FUS-DDIT3, pan-RUNX, FOSL2, and BET proteins across cis-regulatory regions of the genome was annotated as below. Peaks that localized within -1,000 bp to +200 bp from the TSS were defined as promoter-bound; those present inside either SEs or typical enhancers were defined as enhancer-bound; remaining peaks were considered as ''other''.
For RNA-seq analysis, Paired-end reads were pseudo-aligned and quantified to Ensemble (hg19; version 88) transcripts using kallisto program. Kallisto results were imported into DESeq2 using tximport Bioconductor package. Differential gene expression analysis was performed using DESeq2 with lfcThreshold argument set to 0.1. Expression values were calculated in terms of FPKM for every gene with DESeq2::fpkm function. Gene Set Enrichment Analysis (GSEA) was performed on all active genes (mean FPKM > 0.5). Transcriptome data for DDLPS samples of Sarcoma cohort (TCGA-SARC, N = 57) were downloaded using TCGABiolinks Bioconductor package via GDC (data freeze from Dec-2017).
Gene Expression Matrix files for microarray dataset GSE21122 were downloaded using GEOquery Bioconductor package. Gene expression for probes mapping to same genes were averaged to get gene level expression for downstream analysis.
The detailed references can be found in the supplemental methods.

For manuscripts utilizing custom algorithms or software that are central to the research but not yet described in published literature, software must be made available to editors/reviewers upon request. We strongly encourage code deposition in a community repository (e.g. GitHub). See the Nature Research guidelines for submitting code & software for further information.

## Data

Policy information about availability of data

All manuscripts must include a data availability statement. This statement should provide the following information, where applicable:

- Accession codes, unique identifiers, or web links for publicly available datasets
- A list of figures that have associated raw data
- A description of any restrictions on data availability

ChIP-seq data of adipocytes (GSE59703; sequence length = 50) and mesenchymal stem cells (GSE16256; sequence length = 36) , and microarray dataset (GSE21122) were from GEO. Transcriptome data for DDLPS samples of Sarcoma cohort (TCGA-SARC, N = 57) were downloaded from GDC (data freeze from Dec-2017). RNA-seq and ChIP-seq data generated in this study have been deposited in NCBI GEO and are available under accession GSE111254. Source data of all WB blot in the figures, and the raw data underlying all reported averages in graphs and heatmaps are provided as a Source Data file.

# Field-specific reporting

Please select the best fit for your research. If you are not sure, read the appropriate sections before making your selection.

☒ Life sciences ☐ Behavioural & social sciences ☐ Ecological, evolutionary & environmental sciences

For a reference copy of the document with all sections, see nature.com/authors/policies/ReportingSummary-flat.pdf

# Life sciences study design

All studies must disclose on these points even when the disclosure is negative.

| | |
|---|---|
| Sample size | Sample sizes were not predetermined statistically. For animal works, minimum sample sizes for individual experiments were determined not on the basis of a statistical method, but according to experience ($n \geq 7$). |
| Data exclusions | No data was excluded. No animal was excluded from in vivo experiments. |
| Replication | All attempts at replication of reported biological assays were successful. |
| Randomization | No specific randomization method was used to allocate mice into different treatment groups. |
| Blinding | The investigators were not blinded to allocation during experiments or outcome assessment. |

# Reporting for specific materials, systems and methods

## Materials & experimental systems

| n/a | Involved in the study |
| --- | --- |
| ☐ | ☒ Unique biological materials |
| ☐ | ☒ Antibodies |
| ☐ | ☒ Eukaryotic cell lines |
| ☒ | ☐ Palaeontology |
| ☐ | ☒ Animals and other organisms |
| ☐ | ☒ Human research participants |

## Methods

| n/a | Involved in the study |
| --- | --- |
| ☐ | ☒ ChIP-seq |
| ☐ | ☒ Flow cytometry |
| ☒ | ☐ MRI-based neuroimaging |

# Unique biological materials

Policy information about availability of materials

| | |
| --- | --- |
| Obtaining unique materials | Pathologically reviewed human LPS samples were collected from National University Hospital Tissue Repository. The study of human LPS samples was approved by the NUS Institutional Review Board. |

# Antibodies

Antibodies used

Please refer to the Supplementary Table 6: List of antibodies.
Antibody Company Catalog
α-Tubulin Santa Cruz sc-8035
β-ACTIN Sigma-Aldrich A1978
BRD1 Santa Cruz sc-398226
BRD2 Proteintech 22236-1-AP
BRD3 Proteintech 11859-1-AP
BRD3 Bethyl A302-367A
BRD4 Cell Signaling Technology 13440
BRD4 Bethyl A301-985A
CBFB Cell Signaling Technology 62184
CRBN Sigma-Aldrich SAB1407456
CRBN Sigma-Aldrich HPA045910
DDB1 GeneTex GTX100130
DDIT3 Cell Signaling Technology 2895
FLAG Sigma-Aldrich F1804
FOSL2 Santa Cruz sc-604X
FOSL2 Cell Signaling Technology 19967
GAPDH Cell Signaling Technology 2118
GFP MBL 598
H3K27ac Activemotif 39133
H3K27ac Abcam ab4729
H3K4me3 Emdmillipore 04-745
H3K4me1 Abcam ab8895
Histone H3 Cell Signaling Technology 4499
MYC Cell Signaling Technology 5605
p21 Cell Signaling Technology 2947
Pan-RUNX Abcam ab92336
RBX1 Santa Cruz sc-393640
RNA-Pol2 Santa Cruz sc-899X
RUNX1 Santa Cruz sc-365644
SNAI2 Cell Signaling Technology 9585

Validation

All primary antibodies used in this study have been validated by manufacturer. We validated specifically the DDIT3 antibody (Cell Signaling Technology, 2895) to detect the FUS-DDIT3 fusion proteins (Supplementary Figure 3a,b).

# Eukaryotic cell lines

Policy information about cell lines

| | |
| --- | --- |
| Cell line source(s) | Human embryonic kidney cells 293T (HEK293T, U2OS (U-2 OS), and MG63 (MG-63) were from ATCC. MLS402 (MLS 402-91), MLS1765 (MLS 1765-92) and GOT-3 cells were generously provided by Dr Pierre Åman. LP6 and LPS141 cells were provided by Dr Christopher DM Fletcher. T778 and T1000 cells were kind gifts from Dr Florence Pedeutour. MLS402/ET, FU-DDLS-1, and LiSa-2 cells were gifts from Dr Eugenio Erba, Dr Jun Nishio, and Dr Peter Möller, respectively. |
| Authentication | All cell lines were examined by short tandem repeat analysis with the Geneprint 10 System Kit (Promega). U2OS and MG63 were authenticated. All LPS cells used in this study were provided by indicated researchers and were not commercially available. STR profile report confirmed that these LPS cells did not match any known cell lines in the reference databases including ATCC, DSMZ, Riken, JCRB, and KCLB. |

| | |
|---|---|
| Mycoplasma contamination | All cell lines were tested negative for mycoplasma. |
| Commonly misidentified lines (See ICLAC register) | No commonly misidentified cell lines were used. |

## Animals and other organisms

Policy information about studies involving animals; ARRIVE guidelines recommended for reporting animal research

| | |
|---|---|
| Laboratory animals | Female NOD/SCID gamma mice (6- to 8-week-old) were used in this study. |
| Wild animals | This study did not involve wild animals. |
| Field-collected samples | This study did not involve samples collected from the field. |

## Human research participants

Policy information about studies involving human research participants

| | |
|---|---|
| Population characteristics | Pathologically reviewed human LPS samples (2 DDLPS and 2 MLPS tissues) were collected from National University Hospital Tissue Repository. Sample information was de-identified. The study of human LPS samples was approved by the NUS Institutional Review Board. |
| Recruitment | N/A |

## ChIP-seq

### Data deposition

☒ Confirm that both raw and final processed data have been deposited in a public database such as GEO.

☒ Confirm that you have deposited or provided access to graph files (e.g. BED files) for the called peaks.

Data access links
*May remain private before publication.*

RNA-seq and ChIP-seq data generated in this study have been deposited in NCBI GEO and are available under accession GSE111254. https://www.ncbi.nlm.nih.gov/geo/query/acc.cgi?acc=GSE111254

Files in database submission

Sampel name Files
LPS141_Input fastq
LPS141_H3K27ac fastq and BW
LPS141_H3K4me1 fastq and BW
LPS141_H3K4me3 fastq and BW
LPS141_RNAPol2 fastq and BW
LPS141_BRD2 fastq and BW
LPS141_BRD3 fastq and BW
LPS141_BRD4 fastq and BW
LPS141_FOSL2 fastq and BW
LPS141_panRUNX fastq and BW
MLS402_Input fastq
MLS402_H3K27ac fastq and BW
MLS402_H3K4me1 fastq and BW
MLS402_H3K4me3 fastq and BW
MLS402_RNAPol2 fastq and BW
MLS402_DDIT3 fastq and BW
MLS402_BRD4 fastq and BW
MLS1765_Input fastq
MLS1765_H3K27ac fastq and BW
LP6_Input fastq
LP6_H3K27ac fastq and BW
DDLPS_T1_Input fastq
DDLPS_T1_H3K27ac fastq and BW
DDLPS_T2_Input fastq
DDLPS_T2_H3K27ac fastq and BW
MLPS_T1_Input fastq
MLPS_T1_H3K27ac fastq and BW
MLPS_T2_Input fastq
MLPS_T2_H3K27ac fastq and BW

Genome browser session (e.g. UCSC)

N/A

## Methodology

Replicates

All ChIP-seq assays were done once.

Sequencing depth

Sample reads_processed reads_aligned multimapped unique_aligned read_length library
LPS141_panRUNX 46,380,208 36,732,552 7,562,846 29,169,706 50 Single-End
LPS141_BRD2 49,088,839 37,844,395 8,468,499 29,375,896 50 Single-End
LPS141_BRD3 56,464,255 44,177,400 8,853,940 35,323,460 50 Single-End
LPS141_BRD4 50,271,058 39,984,572 7,902,684 32,081,888 50 Single-End
LPS141_H3K27Ac 46,014,214 42,741,454 2,504,532 40,236,922 50 Single-End
LPS141_H3K4me1 48,972,670 44,676,243 3,485,786 41,190,457 50 Single-End
LPS141_H3K4me3 58,174,995 54,927,888 1,819,656 53,108,232 50 Single-End
LPS141_Input 38,835,781 32,824,812 4,573,364 28,251,448 50 Single-End
LPS141_RNAPol2 41,329,451 34,788,539 4,408,849 30,379,690 50 Single-End
MLS402_DDIT3 49,415,732 41,799,511 4,882,088 36,917,423 50 Single-End
MLS402_H3K27Ac 41,441,971 38,467,110 2,300,948 36,166,162 50 Single-End
MLS402_H3K4Me1 46,615,507 41,050,573 4,596,418 36,454,155 50 Single-End
MLS402_H3K4Me3 49,767,029 47,455,007 1,475,842 45,979,165 50 Single-End
MLS402_Input 42,062,923 35,691,225 4,851,811 30,839,414 50 Single-End
MLS402_RNAPol2 41,760,285 35,734,785 4,522,709 31,212,076 50 Single-End
MLS402_BRD4 39,027,102 31,743,152 5,548,064 26,195,088 50 Single-End
MLS1765_Input 37,374,276 31,763,438 4,328,864 27,434,574 50 Single-End
MLS1765_H3K27Ac 42,031,666 38,308,024 3,035,594 35,272,430 50 Single-End
LP6_Input 27,656,266 23,267,697 3,240,372 20,027,325 50 Single-End
LP6_H3K27Ac 23,057,825 20,431,183 2,162,950 18,268,233 50 Single-End
DDLPS_T1_Input 27,284,670 22,810,545 3,298,124 19,512,421 50 Single-End
DDLPS_T1_H3K27Ac 30,531,579 27,637,048 2,355,268 25,281,780 50 Single-End
DDLPS_T2_Input 26,146,950 21,940,492 3,129,619 18,810,873 50 Single-End
DDLPS_T2_H3K27Ac 32,332,930 29,190,637 2,492,461 26,698,176 50 Single-End
MLPS_T1_Input 40,461,645 33,452,116 5,375,885 28,076,231 50 Single-End
MLPS_T1_H3K27Ac 28,464,559 24,161,733 2,663,250 21,498,483 50 Single-End
MLPS_T2_Input 26,146,950 21,940,492 3,129,619 18,810,873 50 Single-End
MLPS_T2_H3K27Ac 32,332,930 29,190,637 2,492,461 26,698,176 50 Single-End

Antibodies

Please refer to the Supplementary Table 6: List of antibodies.

Peak calling parameters

peak caller: macs2

```
macs2 callpeak -t foo.bam -c input.bam -f auto -g hs --keep-dup 1 --outdir sample_foo \
        --name sample_foo --bdg --SPMR --nomodel --extsize 200 -q 0.01
```

Data quality

Sample Peak_number above_5FC percent_above_5FC
LPS141_panRUNX 2,733 2,055 75.19%
LPS141_BRD2 11,764 6,717 57.10%
LPS141_BRD3 9,496 5,581 58.77%
LPS141_BRD4 19,241 10,227 53.15%
LPS141_H3K27Ac 74,574 56,690 76.02%
LPS141_H3K4me3 25,144 22,172 88.18%
LPS141_RNAPol2 29,524 18,070 61.20%
MLS402_DDIT3 41,850 32,423 77.47%
MLS402_H3K27Ac 63,063 46,256 73.35%
MLS402_H3K4Me3 22,216 20,170 90.79%
MLS402_RNAPol2 32,638 16,561 50.74%
MLS402_BRD4 9,214 2,399 26.00%
MLS1765_H3K27Ac 73,006 49,878 68.30%
LP6_H3K27Ac 67,696 42,632 63.00%
DDLPS_T1_H3K27Ac 81,587 61,907 75.90%
DDLPS_T2_H3K27Ac 64,221 41,691 64.90%
MLPS_T1_H3K27Ac 74,386 41,698 56.10%
MLPS_T2_H3K27Ac 89,671 58,498 65.20%

Software

Software (Purpose)
Bowtie (Alignment)
Picard (Marking PCR duplicates)
Macs2 (Peak calling)
bedgraphtobigwig (converting bedgraph files to bigWig)
bwtool (Processing bigWig files)
deeptools (Plotting)

## Flow Cytometry

### Plots

Confirm that:

- ☒ The axis labels state the marker and fluorochrome used (e.g. CD4-FITC).
- ☒ The axis scales are clearly visible. Include numbers along axes only for bottom left plot of group (a 'group' is an analysis of identical markers).
- ☒ All plots are contour plots with outliers or pseudocolor plots.
- ☒ A numerical value for number of cells or percentage (with statistics) is provided.

### Methodology

| | |
|---|---|
| Sample preparation | Cells receiving indicated treatment were trypsinized, washed and fixed in 70% ethanol at 4℃. Cells were washed with cold PBS, resuspended in propidium iodide solution containing RNase A, and incubated at 37℃ for 30 min. After measurement by LSR II Flow Cytometer System (10,000 event per acquisition), cell cycle distribution was analyzed by FlowJo software. |
| Instrument | LSR II Flow Cytometer System (BD Biosciences) |
| Software | FlowJo software |
| Cell population abundance | No cell sorting was employed. |
| Gating strategy | Using the FSC/SSC gating, debris was removed by gating on the main cell population. Figure exemplifying the gating strategy is provided in the Supplementary Information. |

☒ Tick this box to confirm that a figure exemplifying the gating strategy is provided in the Supplementary Information.
